# PM21-particle stimulation augmented with cytokines enhances NK cell expansion and confers memory-like characteristics with enhanced survival

**DOI:** 10.3389/fimmu.2024.1383281

**Published:** 2024-04-22

**Authors:** Jeremiah L. Oyer, Tayler J. Croom-Perez, Md Faqrul Hasan, Javier A. Rivera-Huertas, Sarah B. Gitto, Joanna M. Mucha, Xiang Zhu, Deborah A. Altomare, Robert Y. Igarashi, Alicja J. Copik

**Affiliations:** Burnett School of Biomedical Science, College of Medicine, University of Central Florida, Orlando, FL, United States

**Keywords:** natural killer cell, NK cell therapy, memory-like NK cells, immunotherapy, adoptive cell therapy

## Abstract

NK cell therapeutics have gained significant attention as a potential cancer treatment. Towards therapeutic use, NK cells need to be activated and expanded to attain high potency and large quantities for an effective dosage. This is typically done by ex vivo stimulation with cytokines to enhance functionality or expansion for 10-14 days to increase both their activity and quantity. Attaining a robust methodology to produce large doses of potent NK cells for an off-the-shelf product is highly desirable. Notably, past reports have shown that stimulating NK cells with IL-12, IL-15, and IL-18 endows them with memory-like properties, better anti-tumor activity, and persistence. While this approach produces NK cells with clinically favorable characteristics supported by encouraging early results for the treatment of hematological malignancies, its limited scalability, variability in initial doses, and the necessity for patient-specific production hinder its broader application. In this study, stimulation of NK cells with PM21-particles derived from K562-41BBL-mbIL21 cells was combined with memory-like induction using cytokines IL-12, IL-15, and IL-18 to produce NK cells with enhanced anti-tumor function. The use of cytokines combined with PM21-particles (cytokine and particle, CAP) significantly enhanced NK cell expansion, achieving a remarkable 8,200-fold in 14 days. Mechanistically, this significant improvement over expansion with PM21-particles alone was due to the upregulation of receptors for key stimulating ligands (4-1BBL and IL-2), resulting in a synergy that drives substantial NK cell growth, showcasing the potential for more effective therapeutic applications. The therapeutic potential of CAP-NK cells was demonstrated by the enhanced metabolic fitness, persistence, and anti-tumor function both *in vitro* and *in vivo*. Finally, CAP-NK cells were amenable to current technologies used in developing therapeutic NK cell products, including CRISPR/Cas9-based techniques to generate a triple-gene knockout or a gene knock-in. Taken together, these data demonstrate that the addition of cytokines enhanced the already effective method of ex vivo generation of therapeutic NK cells with PM21-particles, yielding a superior NK cell product for manufacturing efficiency and potential therapeutic applications.

## Graphical Abstract

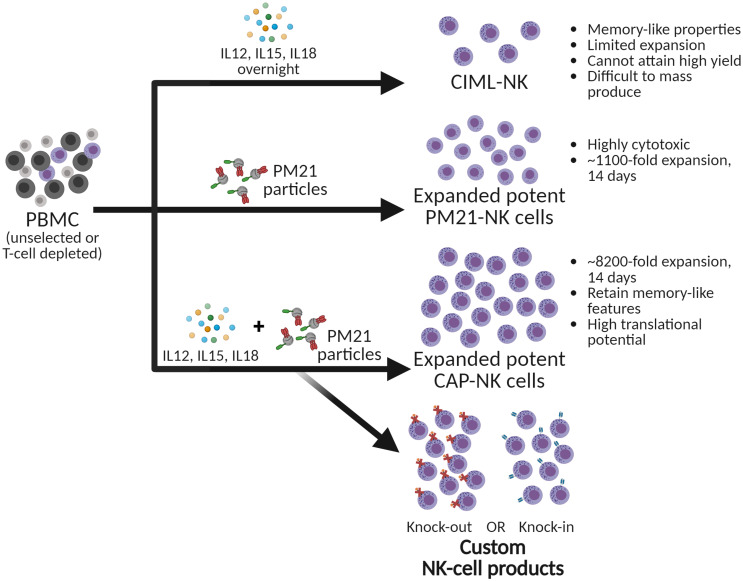

## Introduction

Natural Killer (NK) cells have attracted great interest in the development of cell-based therapies for the treatment of cancer (reviewed in (1–3)). NK cells are a type of innate immune effector cells that detect and destroy virally infected, stressed, or malignant cells without prior sensitization or antigen presentation through their repertoire of activating and inhibitory receptors (reviewed in (4)). They also recognize and kill cells opsonized with IgG1 antibodies that bind FcγR on the NK cells and induce antibody-dependent cell-mediated cytotoxicity (ADCC), a mechanism important for the efficacy of many therapeutic antibodies such as trastuzumab, rituximab or cetuximab. Importantly, NK cells also recruit and prime the innate and adaptive arms of the immune system through the release of chemokines and pro-inflammatory cytokines for a coordinated and effective anti-tumor response (5–8).

One of the notable advantages of NK cells is their potential as a readily available cell therapy. NK cells do not cause graft-vs-host disease (GVHD) when derived from allogeneic sources (9–11). Additionally, when combined with bispecific engagers or engineered with chimeric antigen receptors (CARs), NK cells have been shown to be effective and safe, without the neurotoxicity or cytokine release syndrome commonly associated with CAR-T cell therapies (12–14). However, the success of cell-based therapies is influenced by the manufacturing strategy, including the type of stimulation used to activate and/or expand NK cells, which directly affects NK cell function, their ability to home to specific sites in the body, their persistence after being infused into patients, and their potential for scalable commercialization toward the future standard of care.

Toward the goal of developing a method for the production of an NK cell therapeutic, we have devised PM21-particles that activate and expand NK cells. These PM21-particles are a derived cell-free membrane preparation from K562-mbIL21-41BBL feeder cells (FC21) that were originally developed by Lee and co-workers (15). The safety and effectiveness of NK cells expanded with the K562-mbIL21-41BBL mentioned above have been demonstrated in the context of hematological malignancies in both relapse/refractory (16) and stem cell transplant settings resulting in significantly improved outcomes (13, 17, 18). However, the use of feeder cells poses potential safety risks due to the use of tumor-derived material (e.g. potential to inject proliferation competent cells, genetic material, etc.) and logistical challenges (e.g. storage and handling under GMP) hindering therapeutic development, requiring extensive testing and assessment prior to release of the product to patients (19, 20).

NK cell expansion with PM21-particles (21, 22) allows for comparable expansion to that observed with feeder cells while avoiding the associated safety risks. PM21-particles stimulate both *in vitro* and *in vivo* NK cell expansion in NSG mice (22). NK cells expanded by PM21-particles (PM21-NK cells) highly express FcγR and CD62L as well as activating receptors such as NKp46 and NKG2D (22) and have increased IFNγ and TNFα production in response to stimulation by cytokines and SKOV-3 cells (23). They are highly cytotoxic and exhibit robust anti-tumor activity *in vitro* against multiple cell lines (23–25) and have improved tumor control *in vivo*, including in an ovarian PDX model (24). A very important aspect for clinical translation is the feasibility of robust cryopreservation to enable off-site manufacturing and clinical distribution. PM21-NK cells can be viably cryopreserved without loss of function for off-the-shelf use (24). These NK cells are now undergoing early-stage clinical studies (NCT05712278, NCT05115630, NCT04220684, NCT05726682).

While the PM21-NK cells and FC21-NK cells are stimulated via the IL-21R/STAT3 and 4-1BB pathways (26), other cytokines have been utilized to stimulate NK cells that confer activation by complementary or overlapping pathways. Induction of memory-like properties in NK cells has been shown to enhance NK cell function and persistence. The use of cytokines IL-12, IL-15, and IL-18 to generate cytokine-induced memory-like NK cells (CIML-NK cells) has been used with success to generate individualized products for clinical applications. CIML-NK cells were first described in the context of murine models where splenic NK cells from *Rag1* ^−/−^ mice donors were cultured overnight with high-dose, IL-12, IL-18, and IL-15, and then transferred into *Rag1* ^−/−^ mice recipients (27). These adoptive NK cells proliferated *in vivo* and responded more robustly to reactivation 1-3 weeks later, producing more IFN-γ upon restimulation with cytokines IL-12/IL-15 or activating receptor ligation (27). These murine CIML-NK cells were shown to persist and retain long-lived memory-like responses and prolong the survival of RMA-S tumor-bearing mice (28, 29).

Human NK cells were also found to exhibit cytokine-induced memory-like responses (30). Human CIML-NK cells had enhanced IFN-γ production upon restimulation with cytokines or K562 leukemia tumor targets (30). In pre-clinical studies, these human CIML-NK cells controlled leukemia better and prolonged the survival of xenografted NSG mice (31) and subsequent studies demonstrated their effectiveness against multiple tumor types (32–35). CIML-NK cells have also been used in clinical trials for patients with relapsed or refractory AML (31) and have shown persistence in patients (36, 37) with enhanced functional responses when re-stimulated with leukemia targets (31). Several more clinical trials evaluating the safety and therapeutic effects of CIML-NK cells against several different malignancies are ongoing (reviewed (38)). However, there are limitations in their application. While the initial results demonstrate the clinical utility of CIML-NK cells for the treatment of cancer, the CIML method does not result in high expansion of NK cells and relies on a single allogeneic product made for each patient, limiting the widespread therapeutic utility of CIML-NK cells. Robust, safe, and cost-effective methods for expanding primary NK cells are required for therapeutic development (39, 40). Methods that both stimulate NK cell memory-like properties and support a robust and reproducible large-scale ex vivo expansion of NK cells would greatly improve their clinical utility.

Here, we tested if the PM21-based NK cell expansion method could be further enhanced by the inclusion of cytokines IL-12, IL-15, and IL-18 to further improve the functionality of PM21-NK cells.

## Materials and methods

### Cell culture

**Figure 1:**
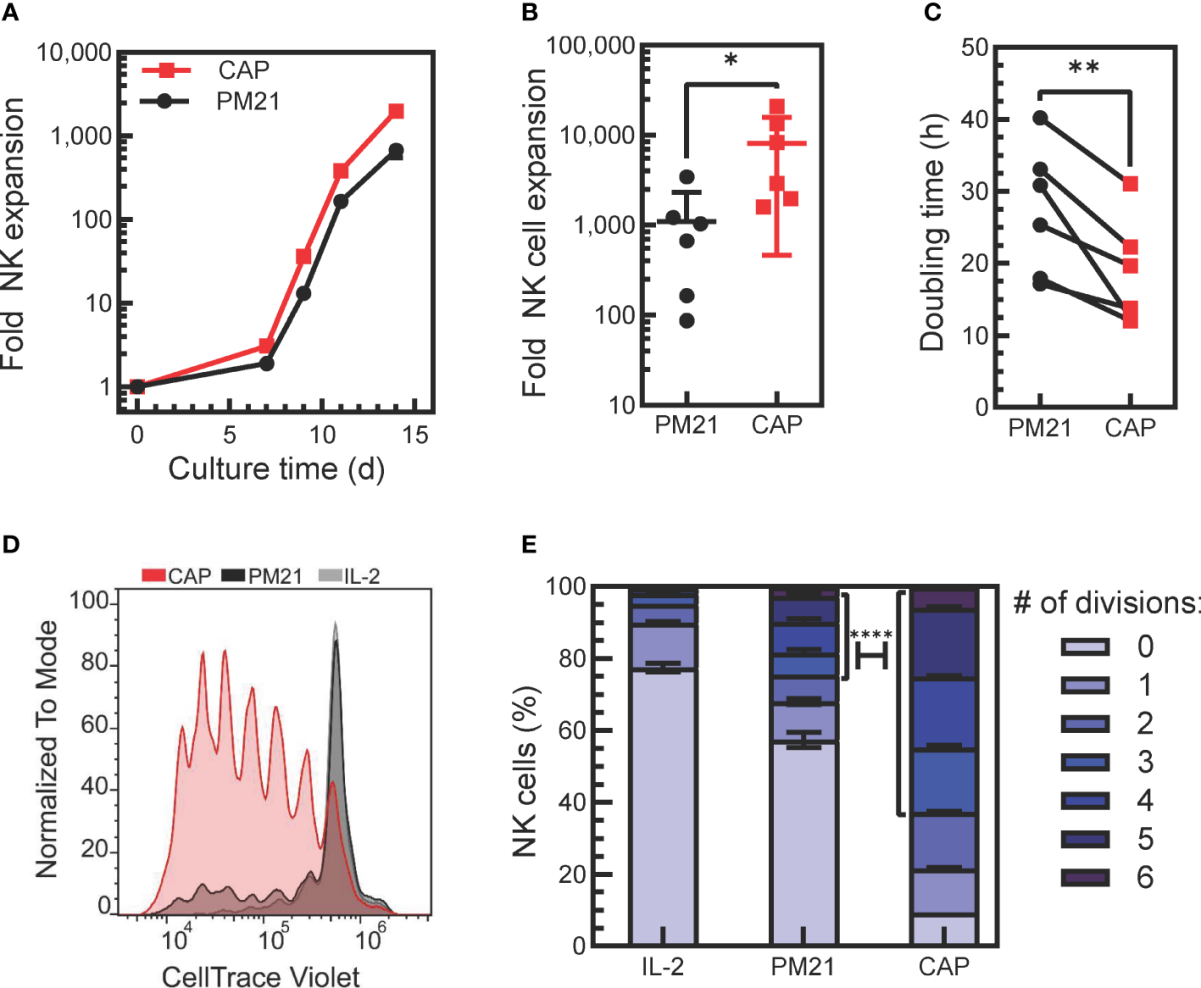
Application of PM21-particles to cytokine-preactivated PBMC resulted in robust expansion of NK cells. **(A)** Representative plot of donor-matched NK cell expansions using PM21-particles to stimulate the expansion of NK cells from either PBMC (PM21, black circles) or PBMC that were preactivated O/N with cytokines IL-12, IL-15, IL-18 (CAP, red squares). **(B)** Cumulative comparison of day 14-fold expansion of NK cells from cultures set up as in A (n=6 donors, each average of duplicate). **(C)** Plot of NK cell division doubling time on day 9 of culture (n=6 donors, each average of duplicate). **(D)** T-cell depleted PBMC labeled with CT violet were either cultured with 100 U/mL IL-2 only as control (IL-2, gray line, light gray fill) or were cultured with PM21-particles with (CAP, red) or without IL-12/15/18 (PM21, black line, dark gray fill) without washing out cytokines. Dye dilution in NK cells was monitored by flow cytometry to assess proliferation, and a representative histogram overlay from day 5 of culture is shown. **(E)** Data on **(D)** were fitted using FLOWJo proliferation tool to determine the number of cells in each generation and results were plotted as a bar graph depicting the percent of NK cells on day 5 of culture that have undergone 0-6 divisions for each NK cell expansion method as described in D (n=2 donors). A χ2 test was used to determine significantly more CAP-NK completed ≥ 3 divisions compared to PM21-NK cells. p values are shown as * if p<0.05, ** if p<0.01, **** if p<0.0001.

PBMC were used as a source for NK cells in all experiments and were obtained from buffy coats (Leukocyte source) from de-identified, healthy donors (OneBlood, Orlando, FL, USA). PBMC were separated by density gradient using Ficoll-Paque Plus solution (GE Healthcare, Chicago, IL, USA) and cryopreserved. Thawed PBMC were used for all NK cell expansions, either unselected or T-cell depleted after thaw as noted. CSTX-002 cells (K562 cell line expressing 4-1BBL and membrane-bound IL-21) used for the preparation of PM21-particles were provided by Kiadis Pharma, a Sanofi company. For all PM21-NK cells, NK cells were expanded for 14 days with PM21-particles as described previously (21–23). Cells were maintained in SCGM (CellGenix GmbH, Freiburg, Germany) media during the first 7 d of expansion and in the RPMI 1640 (Cytiva, Marlborough, MA, USA) media for the remaining culture period and during all assays. For cytokine and PM21-activated (CAP)-NK cell cultures in **Figures 1A-C**, CAP-NK cells were seeded with 10 ng/mL IL-12 (Peprotech, Cranbury, NJ, USA), 100 ng/mL IL-15 (Peprotech, Cranbury, NJ, USA) and 50 ng/mL IL-18 (MBL International, Woburn, MA, USA) in SCGM (CellGenix GmbH, Freiburg, Germany) media without IL-2 and PM21 for 18 h. After 18 h, cells were washed to remove cytokines and re-seeded at 250,000 NK cells/mL with 100 U/mL IL-2 (Peprotech, Cranbury, NJ, USA) and 200 µg/mL PM21-particles and maintained using the PM21 method described previously. For all other experiments, CAP-NK cells were seeded with IL-2, IL-12, IL-15, IL-18 and PM21-particles in culture media at the above concentrations and left for 5 days. Half-media replacements for both PM21- and CAP-NK cultures were performed starting on day 5 and cells were maintained at 0.25 x 10^6^ NK cells/mL. A schematic depicting the expansion procedures is shown in **Supplementary Figure 1**. For G-Rex® cultures, cells were seeded in G-Rex24 and left untouched for 7 days and then transferred to T-flasks, G-Rex6M, or G-Rex100M and cultured for another 7 days. Glucose was monitored starting day 5. NK cell cryopreservation, post-thaw viability, and post-thaw recovery were conducted as previously described (24). Cancer cell lines K562 (ATCC Cat#HTB-77, RRID : CVCL_0532), A549 (ATCC Cat#CCL-185) and SKOV-3 (ATCC Cat#HTB-77, RRID : CVCL_0532) were maintained according to ATCC recommendations. NucLight Red (NLR) expressing A549 and SKOV-3 cancer cell lines were generated through stable transduction using commercial NucLight Red (NLR) Lentivirus (Sartorius, Göttingen, Germany). K562-GFPLuc cells were generated through stable transduction using GFP Lentivirus prepared in-house (Addgene, Watertown, MA, USA). All cell lines were positively selected via puromycin selection followed by sorting on uniformly positive populations using a BD FACS Aria II. All cells were maintained in a humidified atmosphere at 37 °C supplemented with 5% (vol/vol) CO_2_ in air. Cell lines were routinely tested for mycoplasma (E-Myco Plus Mycoplasma PCR Detection Kit, Bulldog-Bio, Inc., Portsmouth, NH, USA) and authenticated via Human STR Profiling (serviced by ATCC).

### Flow cytometry

The antibodies used for flow cytometry analysis are listed in **Supplementary Table 1**. NK cells were stained with pre-conjugated protein-specific, or the corresponding isotype control, antibodies. All samples were acquired on a Cytoflex (Beckman Coulter, Brea, CA, USA) or Northern Lights 2000 Full Spectrum (Cytek, Fremont, CA, USA) flow cytometer and analyzed with CytExpert (Beckman Coulter, Brea, CA, USA; v2.4) or FlowJo software (v10.6.2). An example of the NK cell gating strategy is shown in **Supplementary Figure 2**.

### Proliferation assays

To measure cell division, T-cell depleted PBMC were washed in DPBS twice followed by incubation with 5 µM Cell Trace Violet (ThermoFisher Scientific, Waltham, MA, USA) in DPBS for 20 min at room temperature. Cells were washed in RPMI + 10% FBS and cultured either with PM21 or CAP methods or with IL-2 alone for 5 days. CT Violet signal was analyzed using a CytoFlex LX flow cytometer. Data was analyzed using the FlowJo cell proliferation tool (BD Biosciences, Ashland, OR, USA). The following formula was used to determine doubling time:


$$\text{doubling time}=\frac{\ln2}{\left(\frac{\ln(N_{t})/\ln\left(N_{0}\right)}{t}\right)}$$

where *N_t_* is the number of cells at time *t*, *N_0_* is the number of cells at 0 h, and *t* = time in h.

### HTRF assay

Intracellular pERK was measured using an HTRF (Homogeneous Time Resolved Fluorescence) assay with the Advanced Phospho-ERK (Thr202/Tyr204) Cellular Kit (Perkin Elmer, Waltham, MA, USA). NK cells expanded with either CAP or PM21 methods for 7 days were seeded at 20 x 10^3^ cells per well in 8 µL RPMI + 10% FBS on a 384-shallow well ProxiPlate (Perkin Elmer, Waltham, MA, USA). 4 µL of IL-2 in RPMI + 10% FBS at varying concentrations was added to wells containing NK cells or to a blank well containing RPMI + 10% FBS alone. The plate was incubated in a 5% CO_2_ incubator at 37 °C for 1 h. 4 µL of lysis buffer provided with the kit was added and allowed 30 min at room temperature for cells to lyse. 4 µL of antibodies pre-mixed according to the kit instructions were added to each well and to wells containing 16 µL of positive control lysate and incubated overnight at 4 °C. The plate was read using a Perkin Elmer EnVision plate reader. The pERK HTRF Ratio was calculated using the following formula:


$$\text{HTRF Ratio}=\frac{\text{Signal at }665\text{ nm}}{\text{Signal at }620\text{ nm}}\times 10^{4}$$

Sigmoidal, 4-Parameter Logistics curves were fit to the dose–response data with GraphPad Prism software (GraphPad Prism v 9.4.1, La Jolla, CA, USA) using a non-linear regression equation of Log[IL-2] vs HTRF Ratio.

### Metabolic assay

Glycolytic rate and mitochondrial respiration were analyzed using a Seahorse XF96 instrument (Agilent Technologies, Santa Clara, CA, USA). NK cells expanded with either PM21 or CAP methods for 7 days were seeded at 1 x 10^5^ cells per well in Seahorse assay medium supplemented with 2 g/L glucose, 1 mM sodium pyruvate, and 2 mM Glutamax on a PDL-coated Seahorse XF96 culture plate. Cells were allowed to settle for 15 min at room temperature before transferring to a 37 °C, CO_2_-free incubator for 1 h. Reagents for the Glycolytic Rate Assay and Mitochondrial Stress Test Assay kits (Agilent Technologies, Santa Clara, CA, USA) were used according to the manufacturer’s instructions. For mitochondrial stress, 1.5 mM Oligomycin, 1 mM FCCP, and 0.5 mM Rotenone/Antimycin A concentrations were used. All conditions were run in triplicate. Data were analyzed using Agilent Technologies WAVE Desktop software.

### RNA sequencing

Donor-matched PM21-NK cells and CAP-NK cells were used as a source for total RNA extractions. Preparation of RNA, sequencing, and analysis were performed as previously described (41). ClusterProfiler was used for preranked Gene Set Enrichment Analysis to determine enriched hallmark-gene sets in CAP-NK cells compared to PM21 NK cells and enrichplot (42) was used to generate dot plots of enriched gene sets.

### Kinetic live-cell imaging cytotoxicity assays

Cancer cell lines stably expressing nuclear red fluorescent protein (NucLight Red; NLR) for tracking were used as target cells and live-cell imaging cytotoxicity assays were performed as previously described (43). Cancer cell monolayers were co-cultured with NK cells at the indicated effector-to-target (E:T) ratios and imaged for 45 h with an IncuCyte^®^ S3 Live-Cell Analysis System (Sartorius, Göttingen, Germany). Target tumor cell growth was tracked over time by red object count per well (ROC). Relative growth of the target cells alone or in the presence of NK cells was determined by normalizing ROC to the value at time 0 (ROC_t_/ROC_t=0_) when NK cells were initially added to determine normalized ROC (nROC). Cytotoxicity (%) was then determined based on the following equation.


$${\text{Cytotoxicity}}^{\text{E}:\text{T}}\left(\%\right)=\left(1-\left(\frac{nROC^{E:T}}{nROC^{T}}\right)\right)\times 100$$

### Annexin-V cytotoxicity assay

NK cells were co-cultured with K562-GFPLuc cells at indicated effector vs target (E:T) ratios for 60 min at 37 °C in a tissue culture incubator. Cells were then centrifuged and stained with an Annexin-V-Pacific Blue antibody, incubated for 15 min at 4 °C and analyzed by flow cytometry. The cytotoxicity was determined based on the absolute amount of Viable Target Cells (GFP^+^/Annexin-V^−^) remaining in each well with effectors (VTC^E:T^) and referenced to average VTC in “target alone” control wells (VTC^T ctrl^).


$$Cytotoxicity^{E:T}\left(\%\right)=\left(1-\frac{VTC^{E:T}}{Avg\;VTC^{Tctrl}\;}\right)\times 100$$

### IFNγ and TNFα production

30 x 10^3^ NK cells were co-cultured with K562 cells for 4 h in the presence of Brefeldin A and Golgi Stop™ at 37 °C. Samples were stained with antibodies against CD3 and CD56 for gating on NK cells. NK cells were then fixed and permeabilized (eBiosciences IC Fixation and permeabilization buffers) and stained for intracellular protein targets (IFNγ and TNFα). Data was acquired by flow cytometry and analyzed by FlowJo software.

### Memory-like functional assays

NK cells expanded for 14 days with either PM21 or CAP methods. NK cells were then cold washed for 2 h as previously described (30) and then cultured either with no cytokines or with 1 ng/mL IL-15 for 7 days. Fold recovery based on viable NK cells was assessed by flow cytometry. Cytotoxicity and IFNγ and TNFα production were determined as described above.

### Mouse model

NSG (NOD-scid IL-2Rγnull, BCBC Cat# 4142, RRID : BCBC_4142) mice were purchased from The Jackson Laboratory (Bar Harbor, ME. USA) and then bred in-house. Mice were housed and handled in accordance with protocols approved by the University of Central Florida Institutional Animal Care and Use Committee, an Association for Assessment and Accreditation of Laboratory Animal Care International (AAALAC) accredited facility.

For measuring the persistence of NK cells, PM21-NK and CAP-NK cells from 3 donors cryopreserved (24) on day 15 of culture were thawed and 1 × 10^7^ viable NK cells were injected into the intraperitoneal cavity of 8- to 12-week-old female NSG mice. No exogenous cytokine support was given during the course of the experiment. Abdominal wash from mice were collected 21 days later and NK cell counts were determined by flow cytometry.

### CAP-NK cell genetic engineering

Triple knockout NK cells were generated by electroporation of Cas9 RNPs into CAP-NK cells on day 7 of expansion, containing gRNA targeting exon 1 (5-ACCCTGATGGGACGTACACT) of the TIGIT gene, exon 2 (5- GCTGTGTTGCACCCAGAACG) of the PVRIG gene, and exon 1 (5- ACTTACCACCGACCATGCAT) of the CD96 gene as previously described (44, 45) using a MaxCyte ATx with program NK-5. CAP-NK cells were electroporated with only Cas9 as a control. mCherry knock-in was performed as previously described (52). Briefly, CAP-NK cells were electroporated with Cas9/RNP targeting AAVS1 on day 7 of culture. Thirty minutes after electroporation, cells were transduced with ssAAV6 to deliver HDR DNA encoding mCherry. mCherry expression was measured 48 h post-transduction and on day 14 of culture by flow cytometry.

### Statistical analysis

Statistical analysis was performed by GraphPad Prism 9.4.1. Paired or unpaired two-tailed Student’s t-tests were used unless noted in the figure legend. All experiments were performed for at least 3 biological replicates. p values less than 0.05 were considered statistically significant. p values are shown as * if p<0.05, ** if p<0.01, *** if p<0.001, **** if p<0.0001.

## Results

### The addition of IL-12, IL-15, and IL-18 with PM21-particles enhanced NK cell proliferation

The ability to generate large quantities of NK cells is required for the development of NK cell therapeutics. Cytokine-induced memory-like NK cells have the desired biological attributes such as enhanced persistence and memory-like responses, but currently, they are generated on a per-patient basis without expansion. To enhance their therapeutic potential, we tested if the addition of IL-12, IL-15 and IL-18 to the procedure with PM21-particles could be used in conjunction to benefit the proliferation and expansion rate of NK cells stimulated with PM21-particles. To do so, healthy donor PBMC were thawed and either cultured directly with PM21-particles or were pre-activated with IL-12/15/18 overnight, washed and then cultured with the PM21-particles. Application of PM21-particles to cytokine-preactivated PBMC resulted in robust expansion of NK cells (**Figures 1A, B**). Cytokine pre-activation combined with the PM21-particle (CAP) stimulation increased the rate and fold of NK cell expansion over 14 days compared to PM21-particle (PM21) stimulation alone. The mean fold expansion on day 14 was 8200-fold (range of 1600-21,000-fold) for CAP-NK cells, significantly more than PM21-NK cells with 1100-fold (range of 90-3400-fold) expansion based on cumulative analysis from multiple donors (p=0.05; n=6 donors, each average of duplicates) (**Figure 1B**). Similarly, the doubling time on day 9 of culture was significantly shorter for CAP-NK cells compared to PM21-NK cells (average of 18 ± 7 h vs 27 ± 9 h, respectively p<0.01; n=6 donors, each average of duplicates) (**Figure 1C**). Based on these results, NK cell expansion by the CAP method is enhanced compared to PM21-particles alone. Although this protocol resulted in robust expansion, for clinical manufacturing, the wash step after overnight activation complicates the manufacturing process. For improved applicability, we tested the expansion method without a cytokine wash step. In this method, T-cell depleted PBMC were cultured with IL-12/15/18 together with PM21-particles and IL-2 at the start of culture without washing out cytokines. To confirm that CAP stimulation in this manner still enhanced the proliferation of NK cells, T-cell depleted PBMC were labeled with CT violet prior to culture and were either stimulated with IL-2 (100 U/mL) only, PM21-particles alone or cytokines and PM21-particles together. The dye dilution in NK cells was monitored by flow cytometry to assess proliferation. CAP-NK cells proliferated more compared to PM21-NK cells or cultures activated with IL-2 alone (**Figure 1D**). On day 5 of culture, 5% of IL-2-activated NK cells, 24% of PM21-NK cells, and 63% of CAP-NK cells had divided 3 or more times, 17% of IL-2-activated NK cells, 19% of PM21-NK cells, and 28% of CAP-NK cells had divided 1-2 times (**Figure 1E**) and 78%, 57% and only 9% of cells remained undivided in the respective cultures. These results showed that a significantly higher percentage of CAP-NK cells completed ≥ 3 divisions on day 5 of culture than that of PM21-NK cells (p<0.0001).

**Figure 2:**
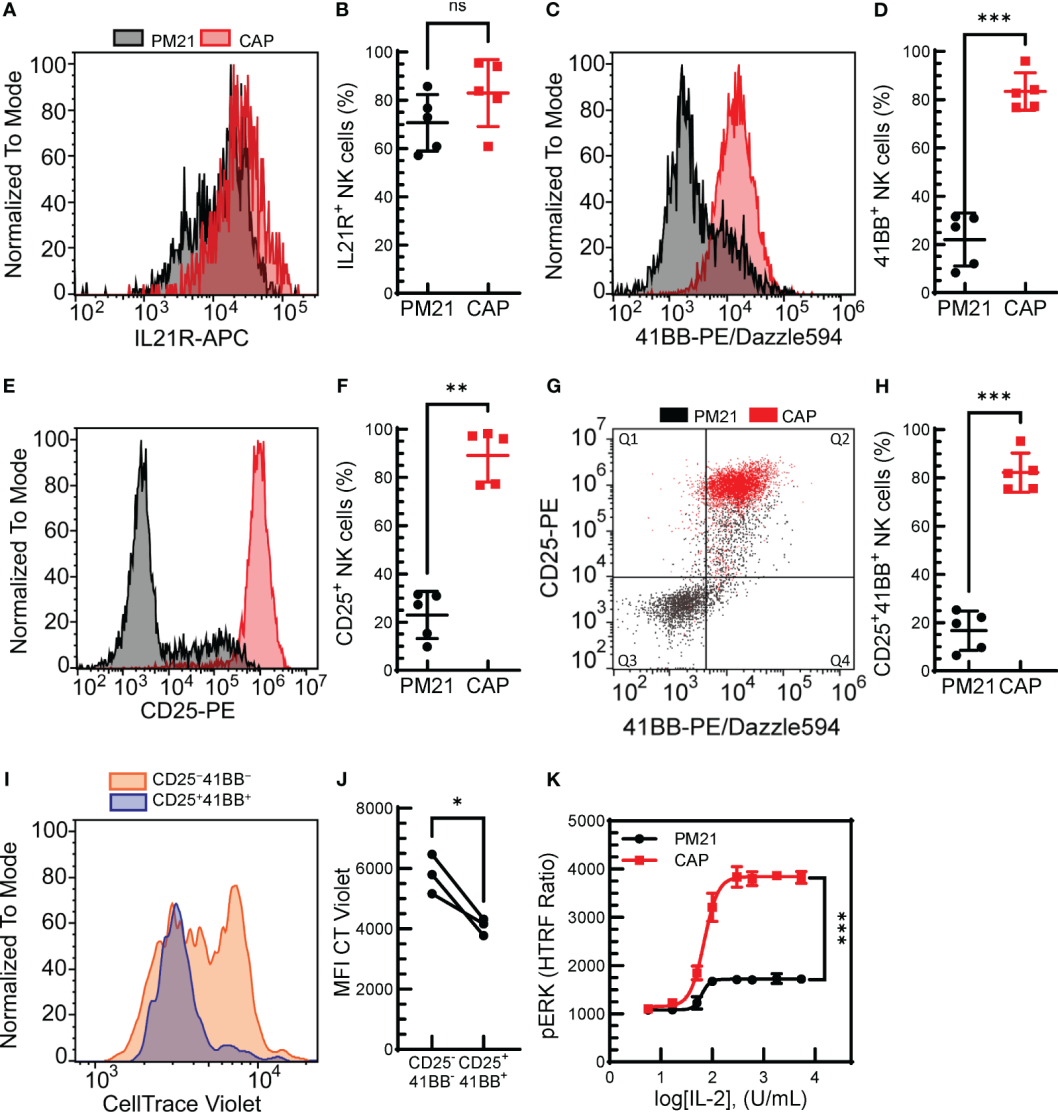
Combined cytokine and PM21-particle activation enhanced the expression of receptors for stimulating ligands. T-cell depleted PBMC were either cultured with IL-12/15/18 together with PM21-particles and IL-2 (CAP, red squares) or were cultured with PM21-particles alone (PM21, black circles). Representative histogram overlay comparing IL21R expression **(A)**, 4-1BB **(C)** and CD25 **(E)** on NK cells from days 5-6 of donor-matched cultures. Cumulative comparison of the percent of IL21R^+^ **(B)**, 4-1BB^+^ **(D)**, and CD25^+^ **(F)** NK cells on day 5 of CAP and PM21-NK cell cultures (n=5 donors, each average of duplicate). **(G)** Representative flow cytometry dot plot overlay of CD25 and 4-1BB expression on NK cells and **(H)** the percent of CD25^+^4-1BB^+^ NK cells on day 5 of CAP and PM21 cultures (n=5 donors, each average of duplicate). **(I)** Representative histogram overlay of CT violet staining remaining in NK cells after 5 days of PM21 culture comparing CD25^+^4-1BB^+^-NK cells (blue) to CD25^−^4-1BB^−^-NK cells (orange). **(J)** cumulative CT violet MFI for the two NK populations after 5 days (n=3 donors, each average of duplicate). **(K)** IL2- concentration-dependent curves for CAP-NK cells and PM21-NK cells as measured by pERK HTRF assay after 1 h exposure to IL-2 at the indicated concentrations (n=1 donor in triplicate) p values are shown as 'ns' if not significant, * if p<0.05, ** if p<0.01, *** if p<0.001.

The initial results indicated that not only can the PM21-particle platform be applied to the expansion of CIML NK cells, but the combined method also results in a synergistic effect on NK cell proliferation. To understand the mechanisms causing the enhanced proliferation of CAP-NK cells after PM21 stimulation compared to resting cells, receptors for the main stimulating molecules, IL-21, 4-1BBL and IL-2 were analyzed on NK cells after short-term (5 days) stimulation with either PM21-particles alone or CAP. The percent of NK cells expressing IL-21R on day 5 was high for both CAP- and PM21-NK cultures, 83 ± 14% and 71 ± 12% IL-21R^+^ NK cells (n=5 donors, each average of duplicates), respectively, and not significantly different (**Figures 2A, B**). However, the percent of CAP-NK cells expressing 4-1BB was significantly increased compared to PM21-NK cells (83% ± 8% vs 22% ± 11%; p=0.0005, n=5 donors, each average of duplicates, **Figures 2C, D**) as well as the fraction of CD25 (IL-2Rα) expressing cells (89% ± 11% vs 23% ± 10%; p=0.001, n=5 donors, each average of duplicates, **Figures 2E, F**). Furthermore, CAP-NK cells dually expressed CD25 and 4-1BB and had significantly more CD25^+^4-1BB^+^ NK cells than PM21-NK cells (82 ± 8% vs 17% ± 8%; p=0.0001, **Figures 2G, H**). To confirm the CD25^+^4-1BB^+^ NK cell population was more proliferative than the CD25^−^4-1BB^−^ NK cell population, a CT Violet dye dilution assay was performed. CT violet intensity was compared between CD25^+^4-1BB^+^ NK cells (**Figure 2G**, Q2) and CD25^−^4-1BB^−^ NK (**Figure 2G**, Q3) cells on day 5 of culture by flow cytometry. The CD25^+^4-1BB^+^ NK cell population had less remaining CT Violet dye than the CD25^−^4-1BB^−^ NK cells, with the MFI significantly decreased across multiple donor-matched pairs (p=0.04, n=3 donors, each average of duplicates) (**Figures 2I, J**). To assess the effect on downstream signaling, IL-2-mediated MAPK pathway activation in CAP-NK and PM21-NK cells was compared by quantifying IL-2 concentration-dependent phosphorylation of ERK by an HTRF®-based assay from three donor sources. While both CAP- and PM21-NK cells had concentration-dependent increases in pERK HTRF signal with a log[EC_50_] of 1.8 U/mL, the maximum production of pERK was significantly higher in CAP-NK cells compared to PM21-NK cells (p<0.001); CAP-NK cells reached a top HTRF ratio of 3800, 95% CI [3751 to 3936], whereas PM21-NK cells topped out at 1700, 95% CI [1673 to1760] (**Figure 2K**). In summary, the PM21-particle platform can be applied to cytokine-preactivated NK cells or simultaneously co-applied with cytokine pre-activation to expand NK cells. The CAP procedure results in faster expansion as compared to PM21-particle alone stimulation due to an increased proliferative potential of the resulting NK cells with greater expression of receptors for stimulating ligands present on the particles.

**Figure 3:**
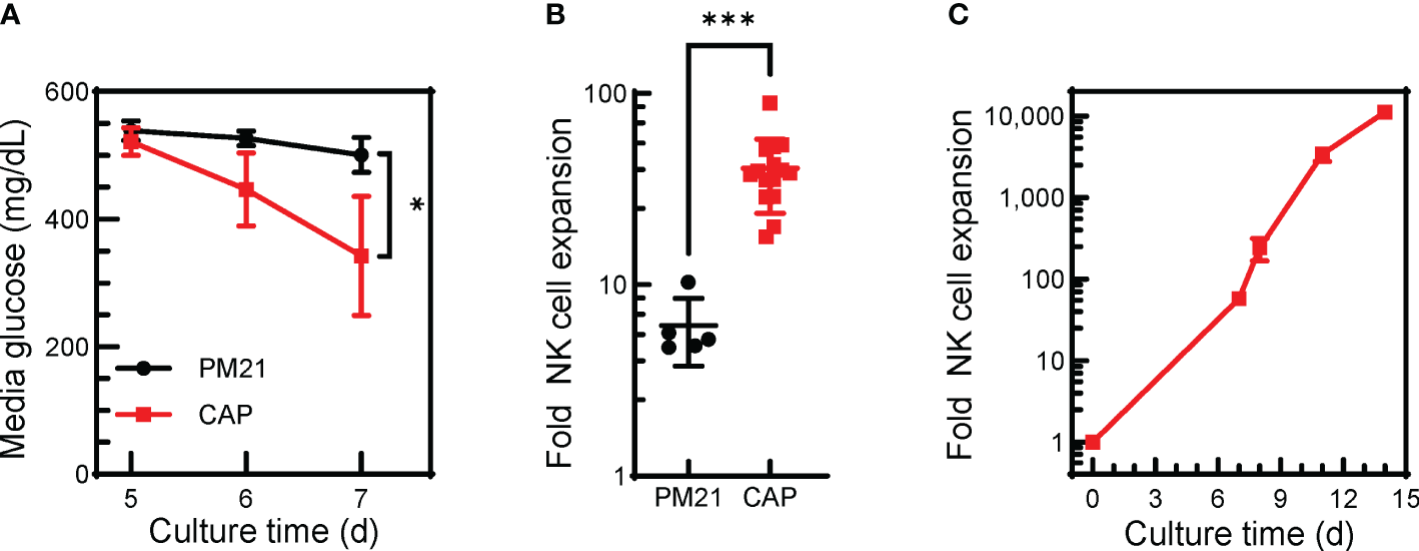
The CAP-NK cell expansion method can be applied in G-Rex® bioreactors. T-cell-depleted PBMC were cultured in a 24-well G-Rex® bioreactor with IL-2 and with either PM21-particles alone or PM21-particles together with IL-12/15/18 (CAP). Cultures were left untouched for 7 days. **(A)** Plot depicting changes in media glucose levels over time of CAP-NK cells (red squares) compared to PM21-NK cells (black circles) (n=5-9 donors). **(B)** Cumulative plot of NK cell expansion on day 7 of CAP NK-cell culture compared to PM21 (n=5-15 donors, each averaged from 1-3 replicates). **(C)** Expansion curve of CAP-NK-cells culture in G-Rex® bioreactors during scale up with sequential transfers to larger size bioreactors (n=average of 2 donors) p values are shown as * if p<0.05, *** if p<0.001.

Finally, to demonstrate the ability of the CAP-NK cell expansion method to be applied to larger scale cultures needed for clinical manufacturing, expansion in G-Rex® bioreactors was tested where IL-12/15/18 with PM21-particles and IL-2 were added together at the start of the culture and left untouched for 7 days. CAP-NK cells grown in G-Rex® bioreactors had lower mean media glucose level on day 7 (343 ± 94 vs 501 ± 27 mg/dL, p<0.01, n=5-9 donors) compared to PM21-NK cells (**Figure 3A**). CAP-NK cells expanded an average 41-fold (range of 18-89-fold) in just 7 days, significantly more compared to an average 6-fold for PM21-NK cells (range of 5-10-fold) (p=0.002, n=5-15 donors, each averaged from 1-3 replicates) (**Figure 3B**). For two donors, CAP cultures were transferred to a larger G-Rex® format after 7 days, and yielded 11,500-fold expansion for donor 1 and 10,800 for donor 2 in 14 days (**Figure 3C**). These results suggest the CAP-NK cell expansion method is amenable to scaling up to large culture platforms.

### CAP-NK cells highly express cell surface markers for NK cell activation

**Figure 4:**
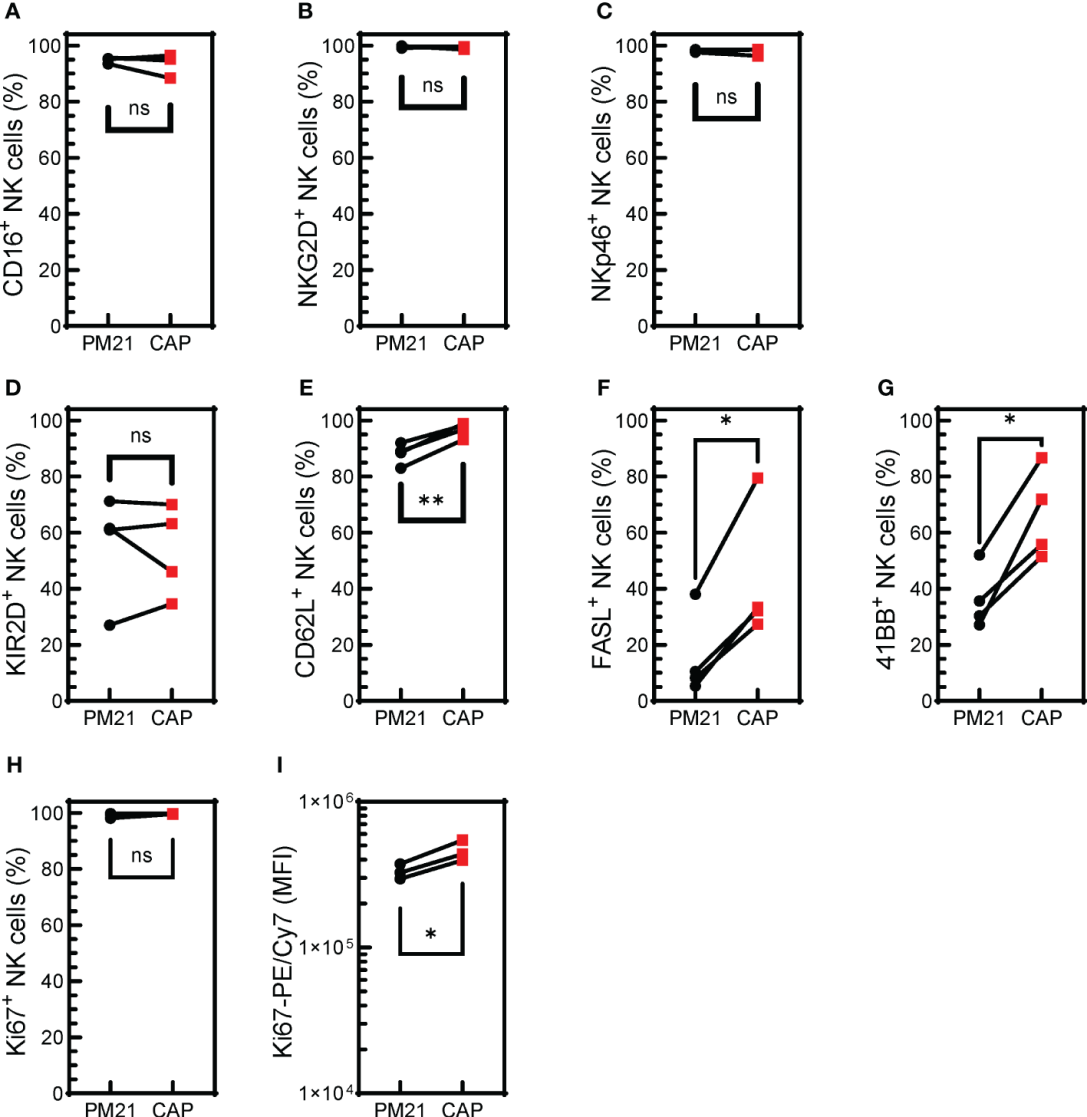
CAP-NK cells highly express activating receptors and markers for NK cell proliferation and activation. T-cell-depleted PBMC were cultured with IL-2 and either PM21-particles alone or PM21-particles together with IL-12/15/18 (CAP) for 14 days. Donor-matched comparisons of surface receptor expression on CAP-NK cells (red squares) and PM21-NK cells (black circles) on day 14 of culture were determined by flow cytometry (n=4 donors). The percent of NK cells expressing CD16 **(A)**, NKG2D **(B)**, NKp46 **(C)**, KIR2D **(D)**, CD62L **(E)**, FAS-L **(F)**, 4-1BB **(G)**, Ki67 **(H)** are shown along with Ki67 mean fluorescent intensity (MFI) **(I)**. p values are shown as 'ns' if not significant, * if p<0.05, ** if p<0.01.

NK cell activation is regulated by a set of activating and inhibitory receptors, and the balance of these inhibitory and activating receptors’ signaling determines NK cell activity (46). The cell surface expression of activating receptors was determined for CAP-NK cells and compared to PM21-NK cells from 4 donors (**Supplementary Figure 3**). CAP-NK cells are phenotypically similar to PM21-NK cells with high expression of activating receptors CD16, NKG2D, and NKp46; all averaged above 94% of NK cells expressing the activating receptors (**Figures 4A–C**). There was no difference in the percent of NK cells expressing KIR2D receptors between donor-matched pairs of CAP- or PM21-NK cells (**Figure 4D**), however, significantly more CAP-NK cells expressed adhesion molecule L selectin (CD62L), associated with polyfunctional NK cells (47) (p<0.004), FasL (p=0.01) and 4-1BB (p=0.01) compared to PM21-NK cells (**Figures 4E-G**). While more than 99% of both CAP- and PM21-NK cells expressed the proliferation marker Ki67, significantly more Ki67 was present in CAP-NK cells, demonstrated by an increase in average mean fluorescent intensity (MFI) from 3.3x10^5^ to 4.6x10^5^ MFI (p<0.03) (**Figures 4H, I**). Taken together, these phenotyping data show CAP-NK cells highly express activating receptors and markers for NK cell proliferation and activation.

### CAP-NK cells have further enhanced effector functions compared to PM21-NK cells

**Figure 5:**
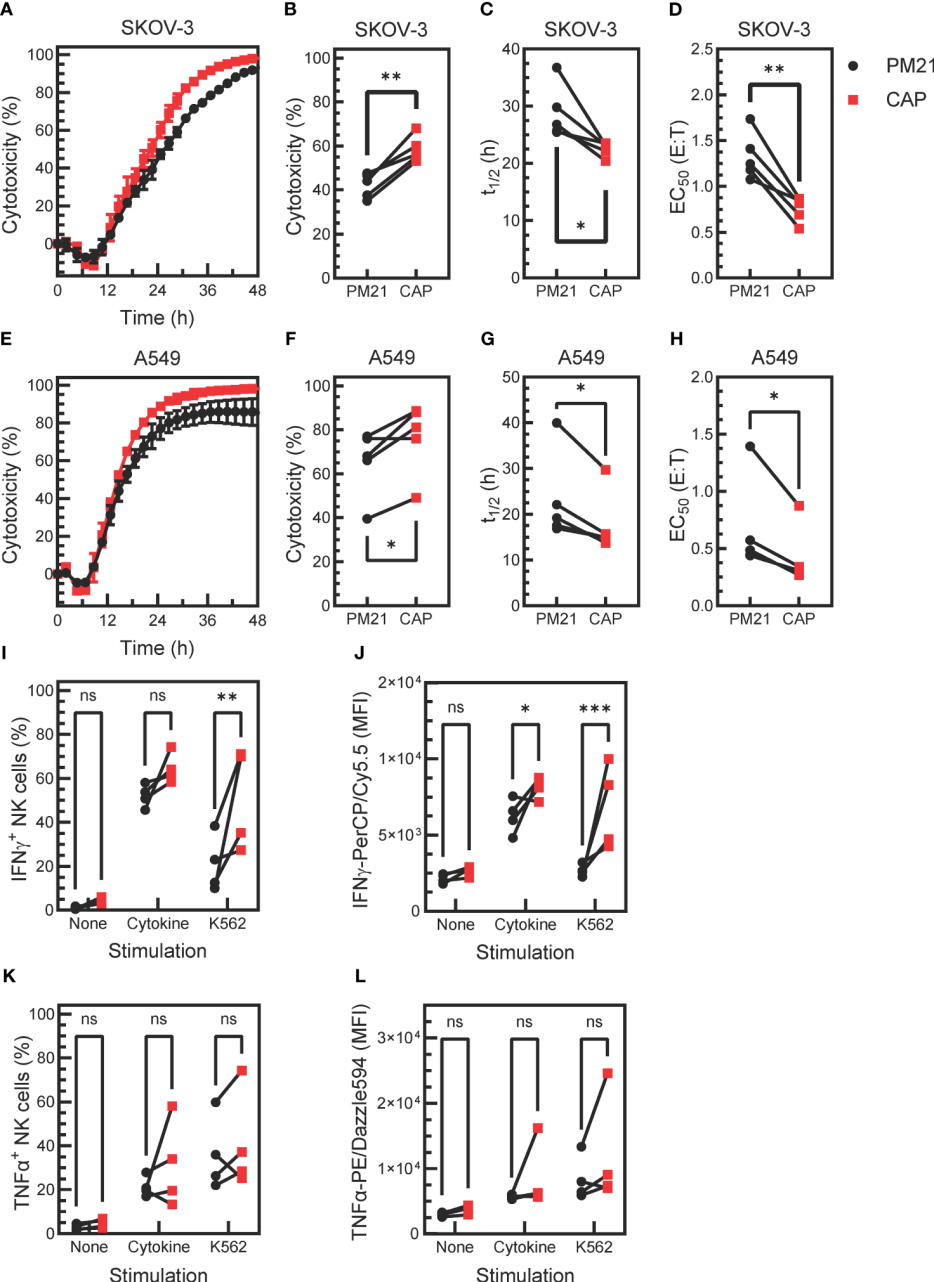
Cytokine activation enhances PM21-NK cell effector functions. T-cell-depleted PBMC were cultured with IL-2 and either PM21-particles alone or PM21-particles together with IL-12/15/18 (CAP) for 14 days. Cytotoxicity of PM21-NK cells (black circles) and CAP-NK cells (red squares) against target cancer cells was determined using an IncuCyte® Live-Cell Analysis System. Representative single-donor time course comparisons of NK cell cytotoxicity at a 1:1 (E:T) against the ovarian cancer cell line SKOV-3 **(A)** and lung cancer cell line A549 **(E)** are shown. Cumulative cytotoxicity **(B, F)**, t_1/2_ at 1:1 E:T **(C, G)**, and EC_50_ at 24 h **(D, H)** comparisons are shown against SKOV-3 and A549, respectively (n=5 donors, each average of duplicates). The percent of IFNγ-producing NK cells **(I)** and MFI of IFNγ-PerCP/Cy5.5 of total NK cells **(J)** that were either unstimulated, stimulated with 10 ng/mL IL-12, 100 ng/mL IL-15 and 50 ng/mL IL-18 or stimulated with K562 tumor cells at 1:1 E:T for 5 h are shown. Similarly, the percentage of TNFα-producing NK cells **(K)** and MFI of TNFα-PE/Dazzle594 of total NK cells **(L)** are shown (n= 4 donors, each average of duplicates for **(I-L)**. p values are shown as 'ns' if not significant, * if p<0.05, ** if p<0.01, *** if p<0.001.

Given that CAP-NK cells had higher proliferation and expression of functional markers, the next question was if CAP-NK cells had further enhanced anti-tumor function compared to PM21-NK cells. To test this, live-cell imaging cytotoxicity assays were performed as previously described (24, 43). Tumor growth in the absence or presence of increasing amounts of NK cells was monitored over time (**Supplementary Figure 4**). Representative kinetic cytotoxicity curves from one donor and summary cytotoxicity, half-killing time, and EC_50_ data from multiple donors are plotted in **Figures 5A–D** against the ovarian cancer cell line SKOV-3 and in **Figures 5E–H** for lung cancer cell line A549. The CAP expansion method enhanced NK cell cytotoxicity against both cancer cell lines compared to PM21 expansion alone, with an average 58 ± 6% vs 42 ± 6% (p=0.003) cytotoxicity against SKOV-3 and 77 ± 16% vs 65 ± 15% (p=0.02) cytotoxicity against A549 at 24 h (1:1 NK cells: target cells, n=5 donors, each average of duplicates). Furthermore, half-killing time (t_1/2_) significantly decreased for CAP-NK cells compared to PM21-NK cells at a 1:1 E:T against SKOV-3 (p=0.03) and A549 (p=0.02) (**Figures 5C, G**). Conecntration-dependent cytotoxicity curves were used to determine EC_50_ values (**Supplementary Figure 4**). CAP-NK cells also had significantly decreased EC_50_ at 24 h compared to PM21-NK cells against SKOV-3 (p<0.005) and A549 (p=0.02) (**Figures 5D, H**). In summary, CAP-NK cells killed target cells faster and required fewer cells, resulting in an overall greater killing capacity.

Production of effector cytokines IFNγ and TNFα was also assessed after stimulating NK cells with cytokines IL12/15/18 or K562 target cells (n=4 donors, each average of duplicates). Unexposed, unstimulated NK cells were used as a negative control. A greater fraction of IFNγ^+^ NK cells was observed in CAP-NK cells as compared to PM21-NK cells in response to cytokine stimulation (64 ± 7% vs 52 ± 5%, p=0.1) and significantly greater in response to K562 cell exposure (51 ± 23% vs 21±%13, p=0.001) (**Figure 5I**). The amount of IFNγ detected in NK cells (based on the MFI of total stained NK cells) was also greater for CAP-NK cells compared to PM21-NK cells, with an average MFI of 8200 ± 700 for CAP-NK cells and 6200 ± 1100 for PM21-NK cells in response to cytokines and of 6800 ± 2800 vs 2700 ± 400 MFI, respectively after K562 cell exposure (**Figure 5J**). The effect on TNFα production was variable and donor-dependent; overall, no significant change was observed (**Figures 5K, L**). Cumulatively, these data show that the CAP-based NK cell expansion method further enhanced PM21-NK cell effector functions.

### CAP-NK cells have increased glycolytic rate and mitochondrial potential compared to PM21-NK cells

**Figure 6:**
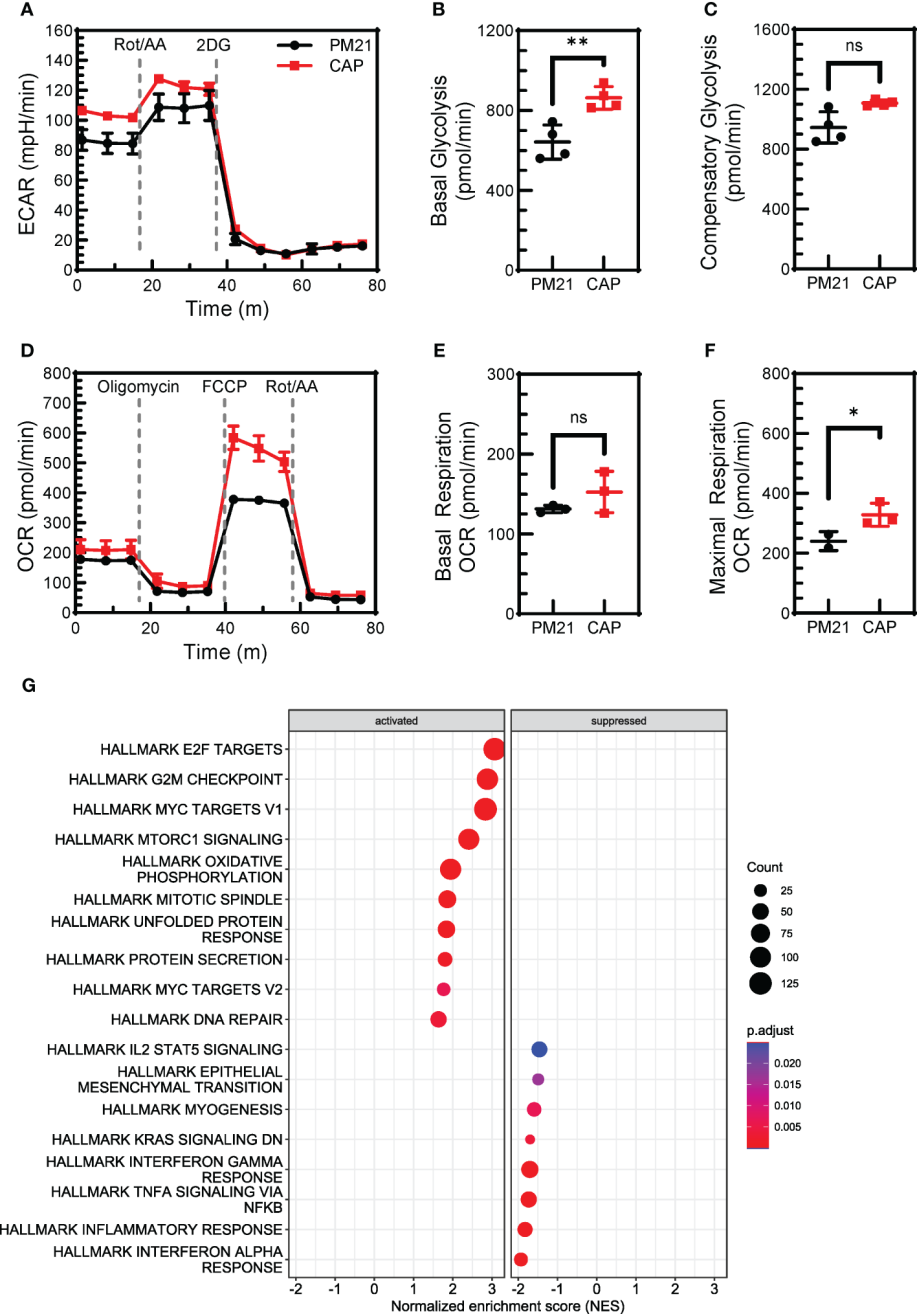
CAP-NK cells have increased cellular metabolism compared to PM21-NK cells. T-cell-depleted PBMC were cultured with IL-2 and either PM21-particles alone or PM21-particles together with IL-12/15/18 (CAP) for 7-14 days. Metabolic profiles of PM21-NK cells (black circles) and CAP-NK cells (red squares) were measured by Seahorse (Agilent Technologies) on day 7 of culture. **(A)** Representative extracellular acidification rate (ECAR) profiles of NK cells from one donor-matched PM21- and CAP-NK cells determined from a Glycolytic Rate Assay. Cumulative graphs comparing basal glycolysis **(B)** and compensatory glycolysis **(C)** of PM21-NK cells and CAP-NK cells (n=3 donors, each average of quadruplicates). **(D)** Representative oxygen consumption rate (OCR) profiles of NK cells from one donor-matched PM21- and CAP-NK cells determined from a Mito Stress Test Assay. Cumulative graphs comparing basal respiration **(E)** and maximal respiration **(F)** of PM21-NK cells and CAP-NK cells (n=1 donor, measured in triplicate. A second donor is shown in **Supplementary Figure 5**). **(G)** Total RNA was extracted from PM21-NK cells and CAP-NK cells after 14 days of culture and sequenced. Dot plots of normalized enrichment score (NES) of hallmark enriched gene sets determined by Gene Set Enrichment Analysis are shown. p values are shown as 'ns' if not significant, * if p<0.05, ** if p<0.01.

Cellular metabolism is essential for proliferation and effector functions of NK cells (reviewed in (48, 49)). Previous studies have shown that IL-12/15/18 exposure increased the glycolytic rate of NK cells (50). CAP-NK cells were found to proliferate more and have enhanced effector function as compared to PM21-NK cells. To determine if CAP-expanded NK cells similarly have improved metabolic fitness as compared to PM21-NK cells, the aerobic glycolysis and OXPHOS were compared for CAP-NK cells to that of PM21-NK cells by real-time measurement of extracellular acidification rate (ECAR) (**Figure 6A**) and oxygen consumption rate (OCR) (**Figure 6D**) on day 7 of culture using the Seahorse assay. CAP-NK cells had significantly increased basal glycolysis compared to PM21-NK cells, 863 ± 56 vs 642 ± 86 pmol/min (p<0.05, n=3 donors, each average of quadruplicates) (**Figure 6B**) as well as trended towards increased compensatory glycolysis, 1108 ± 19 vs 945 ± 104 pmol/min (p=0.06, n=3 donors, each average of quadruplicates) (**Figure 6C**). While there was no change in basal respiration (**Figure 6E**), CAP-NK cells had significantly increased maximal respiration compared to PM21-NK cells; 428 ± 113 vs 297 ± 55 pmol/min, respectively (p<0.01, n= 2 donors, each average of 2-3 replicates) (**Figure 6F**; **Supplementary Figure 5**). To determine if RNA expression of metabolic pathways was augmented in CAP-NK cells, total RNA was extracted from CAP-NK cells and PM21-NK cells, sequenced, and hallmark-gene set enrichment analysis was performed. CAP-NK cells were enriched in both metabolic and cell division hallmark pathways compared to PM21-NK cells, confirming the results of functional analysis (**Figure 6G**). Thus, CAP-NK cells have improved metabolic fitness as compared to PM21-NK cells.

### CAP-NK cells exhibit memory-like properties

**Figure 7:**
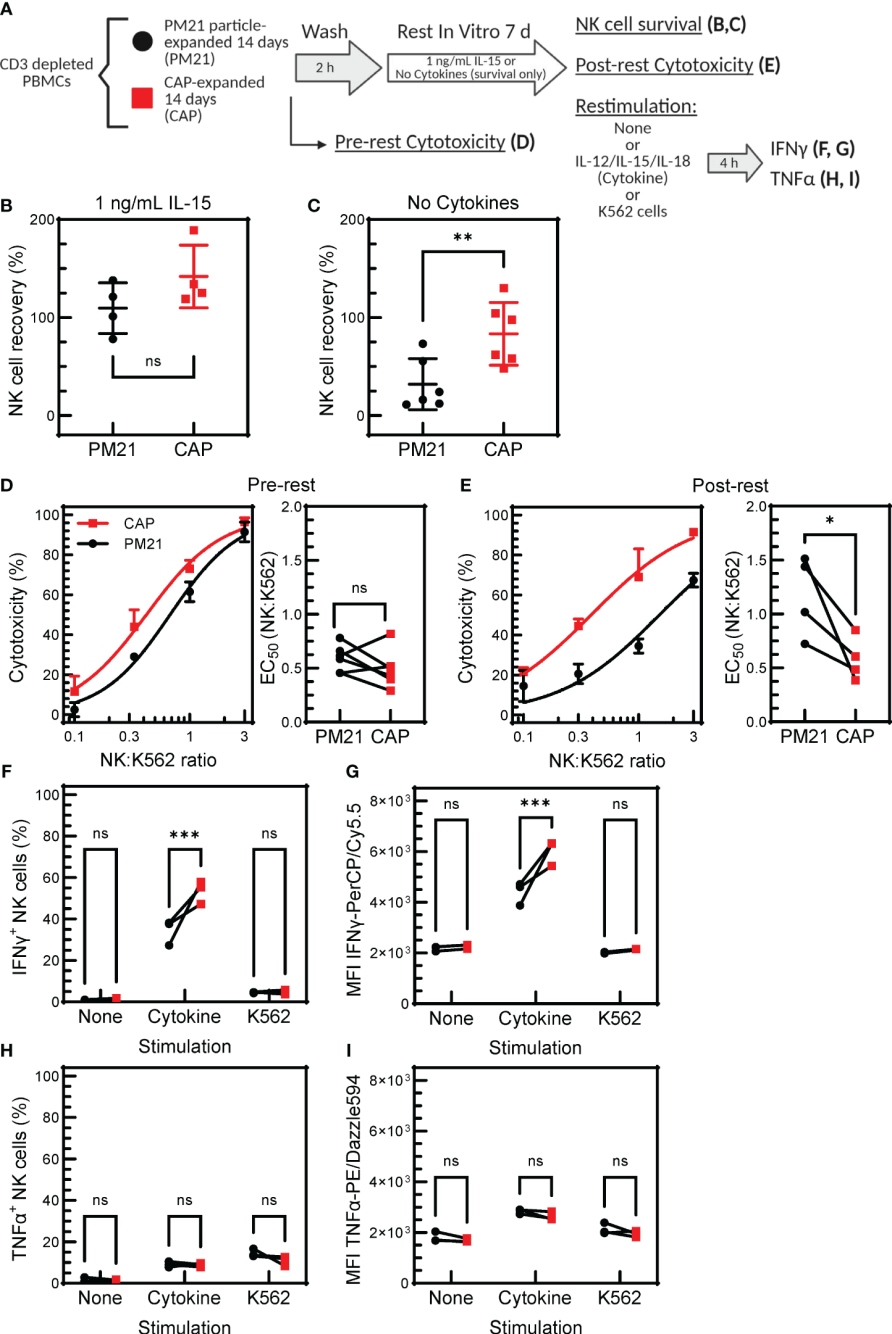
CAP-NK cells exhibit improved memory-like function. T-cell-depleted PBMC were cultured with IL-2 and either PM21-particles alone (PM21, black circles) or PM21-particles together with IL-12/15/18 (CAP, red squares) for 14 days. NK cells were then rested by cold-washing multiple times for 2 h and cultured either with no cytokine support or with 1 ng/mL IL-15 for 7 days prior to analysis. A schematic of the experiment is shown in **(A)**. Dot plots depicting percent of NK cells surviving in culture after 7 d rest with 1 ng/mL IL-15 support **(B)** or no cytokine support **(C)** are shown (n=4-6 donors). Single donor representative concentration-dependent curves across 4 E:T ratios and cumulative EC_50_ (n=4-6 donors, each average of duplicates) against K562 for CAP-NK cells and PM21-NK cells pre-rest **(D)** and post-rest with 1 ng/mL IL-15 cytokine support **(E)** are shown. Effector cytokine production was also measured post-rest. The percent of IFNγ-producing NK cells after rest **(F)** and MFI of IFNγ-PerCP/Cy5.5 **(G)** of NK cells after rest that were either unstimulated, stimulated with 10 ng/mL IL-12, 100 ng/mL IL-15 and 50 ng/mL IL-18 or stimulated with K562 tumor cells at 1:1 E:T for 5 h are shown. Similarly, the percentage of TNFα-producing NK cells after rest **(H)** and MFI of TNFα-PE/Dazzle594 **(I)** are shown (n=3 donors,each average of duplicates) in response to the specified stimulus. p values are shown as 'ns' if not significant, * if p<0.05, ** if p<0.01, *** if p<0.001.

Memory-like properties, an enhanced functional response after an initial pre-activation and return to baseline state (27), have been documented for NK cells in various settings, including after pre-activation with IL12/15/18 (30, 31, 34, 51). Specifically, cytokine-preactivated NK cells have improved persistence and retained enhanced IFNγ-response following a period of rest and reactivation. To determine if CAP-NK cells have memory-like properties, expanded CAP-NK cells were rested for 7 days as previously described and restimulated with either cytokines or tumor targets. NK cell function was assessed before and after rest and compared to PM21-NK cell controls (**Figure 7A**) (30). To determine the survival of NK cells with or without cytokine support during the rest period, NK cells for conditions as specified in **Figure 7A** were extensively cold-washed over a 2 h period to remove all bound and unbound cytokines and then rested for 7 days either with a low concentration of IL-15 or without any added cytokines. The survival was similar for CAP-NK cells or PM21-NK cells when using low-dose IL-15 support during the rest period; both averaged greater than 100% recovery, indicating some expansion was still occurring during rest (**Figure 7B**) (n=4 donors). When no cytokine support was provided during the rest period, an average of 85 ± 35% of CAP-NK cells were recovered compared to 35 ± 25% of PM21-NK cells (**Figure 7C**) (n=6 donors). This indicates that CAP-NK cells were able to survive even without any exogenous cytokine support. Using the CAP- and PM21-NK cells rested for 7 days with low dose IL-15 support, cytotoxicity against K562 cells was measured pre- and post-rest. Concentration-dependent cytotoxicity curves were generated using an Annexin-V flow cytometry-based cytotoxicity assay, and EC_50_ values were determined as previously described (25). No significant difference in cytotoxicity pre-rest was observed between CAP- and PM21-NK cells (EC_50_ of 0.6 ± 0.1 E:T for PM21-NK and 0.5 ± 0.2 E:T for CAP-NK cells) (**Figure 7D**); however, post-rest, CAP-NK cells were more potent compared to PM21-NK cells with an EC_50_ of 0.6 ± 0.2 E:T compared to the reduced capacity observed in PM21-NK cells with an EC_50_ of 1.2 ± 0.4 E:T (**Figure 7E**) (p=0.03, n=4-6 donors, each average of duplicates). Effector cytokine production was also measured post-rest. Significantly more NK cells produced IFNγ after 7 days of rest from CAP expanded cultures than PM21 when comparing donor-matched pairs (p=0.0002, n=3 donors, each average of duplicates) (**Figure 7F**). Total IFNγ as measured by MFI also was significantly greater in CAP- vs PM21-NK cells (p=0.0002, n=3 donors, each average of duplicates) (**Figure 7G**). Minimal IFNγ protein was produced post-rest in response to K562 and no difference between CAP- and PM21-NK cells was observed (**Figure 7G**). Similar to pre-rest TNFα production, no significant differences were observed between CAP- and PM21-NK cells (**Figures 7H, I**) (n=3 donors, each average of duplicates). Taken as a whole, these data demonstrate that CAP-NK cells persist better, are cytotoxic after prolonged rest and exhibit memory-like responses upon restimulation.

### CAP-NK cells demonstrated improved performance *in vivo*

**Figure 8:**
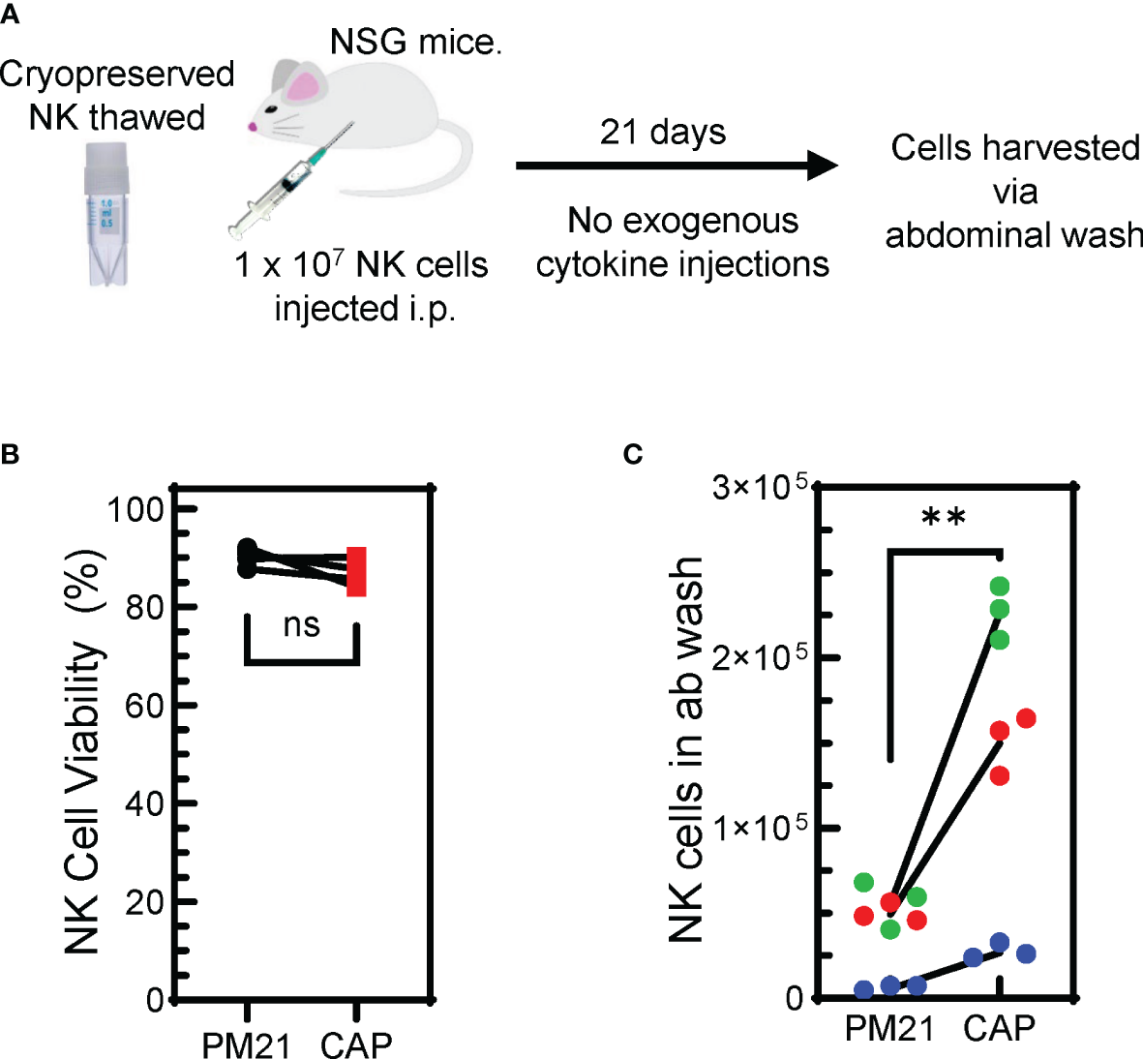
Cryopreserved CAP-NK cells show improved persistence *in vivo.* **(A)** Schematic depicting the *in vivo* persistence experiment. Cryopreserved CAP-NK cells and PM21-NK cells from 3 donor sources were thawed and immediately injected i.p into NSG mice. After 21 days with no exogenous cytokine support, abdominal washes were collected from mice. **(B)** Post-thaw viability of cryopreserved CAP-NK cells (red squares) compared to PM21-NK cells (black circles) prior to injection into NSG mice. **(C)** Post-euthanasia recovery on day 21 of intraperitoneal human NK cells from abdominal wash. Each color represents a different donor source and black lines show donor-matched comparisons of NK cells recovery from mice injected with PM21- or CAP-NK cells. p values are shown as 'ns' if not significant, ** if p<0.01.

In order for CAP-NK cells to be considered as a potentially favorable therapeutic option, the demonstration of their improved persistence and tumor control *in vivo* would be beneficial. Two *in vivo* experiments were conducted to assess this. First, to determine NK cell persistence *in vivo* (**Figure 8A**), PM21-NK and CAP-NK cells from 3 donors cryopreserved (24) on day 15 of culture were thawed. Experiments assessing the amenability of CAP-NK cells to cryopreservation confirmed there is no difference in post-thaw overnight NK cell recovery and post-thaw expression of NKp46, NKG2D, or CD16 between CAP- and PM21-NK cells (**Supplementary Figure 6**). Similarly, for this *in vivo* experiment, the viability after thaw was not different between CAP- and PM21-NK cells and was greater than 85% (**Figure 8B**). NK cells (1 × 10^7^ viable cells) were then injected into the intraperitoneal cavity of 8- to 12-week-old female NSG mice. No exogenous cytokine support was given during the course of the experiment. Abdominal wash from mice was collected 21 days later, and the number of NK cells was determined (**Figure 8C**). CAP-NK cells had significantly increased persistence compared to PM21-NK cells (p=0.005). To confirm anti-tumor activity and the ability to home, *in vivo* anti-tumor activity of CAP-NK cells was compared to PM21-NK cells using an orthotopic leukemia model (**Supplementary Figure 7A**). K562-Luc cells were delivered into the bone marrow via kneecap injections. CAP- or PM21-NK cells were then administered one day later via tail vein injection. After 22 days, with IL-2 injections 3x/week, mice were imaged to determine the presence of K562-Luc tumor cells. One out of five mice treated with CAP-NK cells had a tumor signal compared to 3 of 5 for PM21-NK cell-treated mice. All five mice in the untreated group had tumors present (**Supplementary Figure 7B**). Taken together, these results indicate that CAP-NK cells have increased persistence *in vivo* and are capable of homing to bone marrow and diminishing leukemia burden in mice.

### CAP-NK cells are amenable to genetic modifications

**Figure 9:**
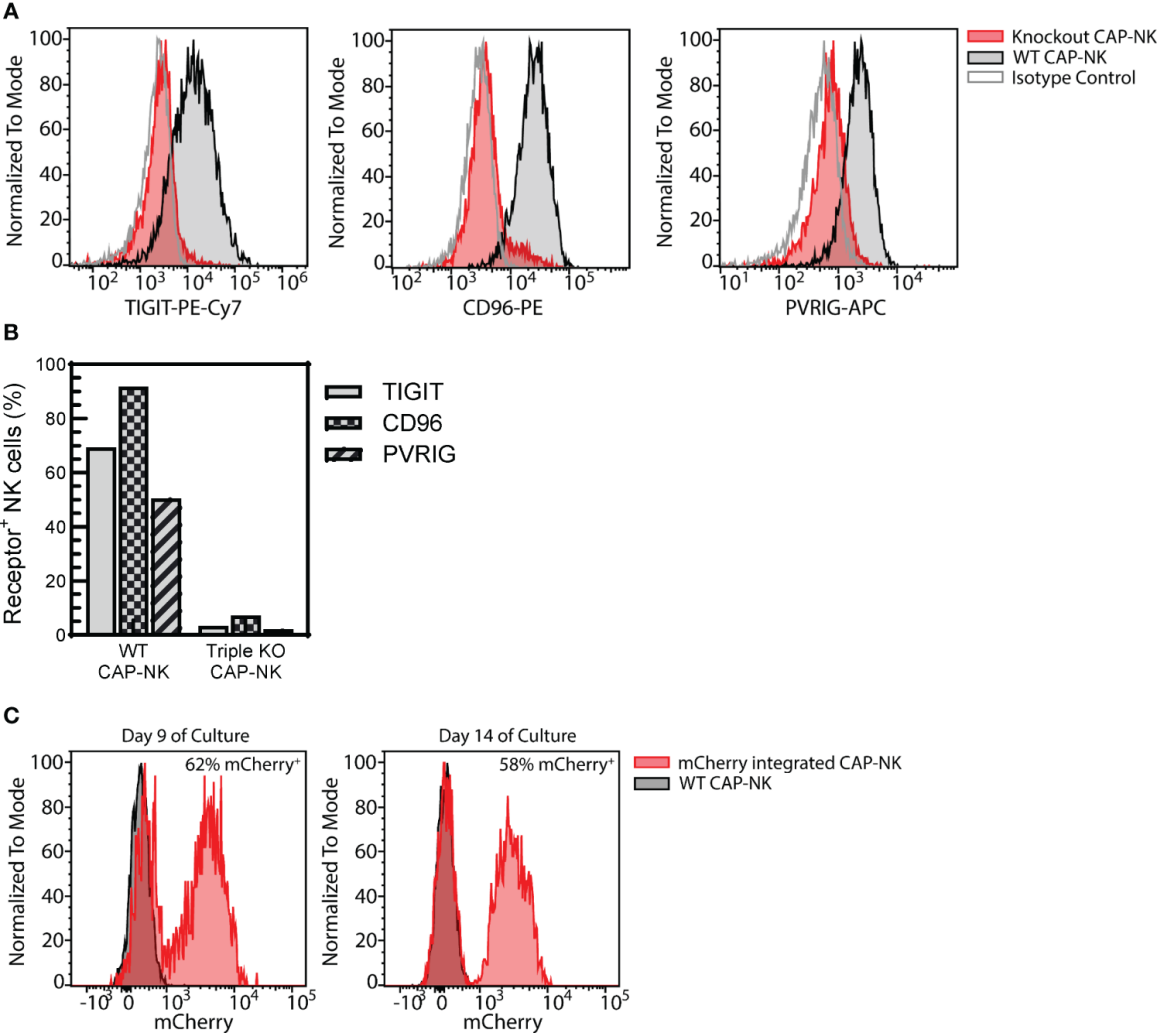
CAP-NK cells are amenable to genetic engineering. CAP-NK cells were electroporated mid-expansion for co-delivery of CRISPR/Cas9 complexes targeting the inhibitory receptors TIGIT, CD96, and PVRIG to abrogate receptor expression. **(A)** Histogram overlays of the expression of each receptor on day 14 of culture in the triple knockout CAP-NK cells (red) compared to WT CAP-NK cells (gray with black outline) and isotype control (white with gray outline). **(B)** The percentage of triple knockout (KO) CAP-NK cells expressing TIGIT (gray), CD96 (checkered), and PVRIG (striped) compared to WT CAP-NK cells. **(C)** Using CRISPR and AAV-based methods, mCherry was inserted into the AAVS1 site in CAP-NK cells. Histogram overlays of transduced CAP-NK cells (red) and non-transduced WT CAP-NK cells (gray, black outline) on day 9 and day 14 of culture.

Genetically engineered NK cell therapy is a promising new therapeutic strategy. A common strategy to enhance NK cells is to genetically knock out the expression of inhibitory receptors. To determine if CAP-expanded NK cells are amenable to this approach, a triple inhibitory receptor knockout was performed using CRISPR/Cas9 targeting as previously described (41). Simultaneous knockout of TIGIT, CD96, and PVRIG could be achieved (**Figure 9A**) with less than 10% of CAP-NK cells expressing each receptor (**Figure 9B**). Alternatively, knock-in strategies, often used for developing CAR-NK cells, are also commonly employed. Using CRISPR and AAV-based methods previously described (52), mCherry was successfully integrated into a targeted AAVS1 site in CAP-NK cells and expression was maintained over time with 62% of NK cells expressing mCherry on day 9 of culture and 58% expressing on Day 14 (**Figure 9C**). These proof-of-concept experiments indicate that CAP-NK cells are amenable to genetic modification.

## Discussion

This study found that stimulating NK cells with cytokines and PM21-particles (CAP) significantly boosted their proliferation compared to stimulation with PM21-particles alone and was amenable to application in a large-scale growth platform. Previous research has shown that pre-activation with interleukins IL-12/15/18 enhanced the expression of CD25 (IL-2Rα), a subunit essential for forming the high-affinity IL-2 receptor IL-2Rαβγ. This complex is crucial for increasing STAT5 phosphorylation in response to IL-2, leading to enhanced production of interferon-gamma (IFN-γ), cytotoxic activity, and cell proliferation (53). CAP-stimulated NK cells not only exhibited higher levels of CD25 but also showed increased expression of 4-1BB and high levels of IL-21R, which are receptors for the ligands present on PM21-particles and for soluble IL-2 used in low concentrations during culture. Moreover, the rise in phospho-ERK (pERK) in response to IL-2 suggests the activation of the MAPK signaling pathway, a key route to NF-κB signaling, which is vital for NK cell proliferation, cytokine production and effector functions (54). Consequently, CAP stimulation led to a significant increase in proliferation, marked by elevated Ki67 expression and the activation of pathways linked to cell division and proliferation. This indicates that the combined stimulation with IL-12, IL-15, IL-18, and PM21-particles could synergistically enhance the generation of CAP-NK cells with improved proliferative and functional capabilities. Particles derived from other K562 feeders cell lines engineered for expression of alternative ligands, such as K562-OX40L-mbIL21, and other feeder cell lines, such as EBV-LCL, could potentially be used in combination with IL12/15/18 cytokine stimulation, however, the ability of these ligands to synergize with cytokine stimulation to provide a proliferative advantage and induce memory-like function would need to be determined.

The studies here show that utilization of multiple synergistically acting cytokines leads to metabolic tunning and enhanced activation characteristics in the CAP-NK cells. Studies have shown that when NK cells are stimulated with interleukins such as IL-12, IL-15, and IL-18, they undergo metabolic reprogramming, which includes increased expression of nutrient transporters like CD71, CD98, GLUT1, and GLUT3 (50). This reprogramming supports increased nutrient uptake essential for their activation and function. Furthermore, these changes lead to a metabolic shift towards glycolysis, characterized by higher rates of glucose uptake and glycolytic activity, even after the initial cytokine stimulation has ceased. This shift towards glycolysis is complemented by changes in mitochondrial activity, indicating an overall increase in metabolic flexibility that supports the heightened effector functions of NK cells during immune responses (49, 55). When NK cells are stimulated with cytokines they have increased metabolism with increased oxidative respiration capacity (56). If these metabolic characteristics are inhibited, memory-like properties of cytokine-activated NK cells are impaired (57). Similarly, CAP-NK cells showed superior metabolic fitness, exhibiting higher glycolytic rates and mitochondrial potential compared to PM21-NK cells. RNA sequencing further indicated that CAP-NK cells have upregulated proliferation and metabolic pathways, including oxidative phosphorylation. The mTORC1 signaling pathway, which plays a pivotal role in NK cell proliferation and metabolism and is essential for IFNγ production, was also upregulated in CAP-NK cells (56, 58) and is likely key to carrying out memory-like responses of cytokine-activated NK cells. The increased metabolic activity and flexibility enable NK cells to meet the energetic and biosynthetic demands of their enhanced effector functions, providing insights into potential metabolism-targeted approaches for modulating NK cell responses in therapeutic settings.

Increased expression of activating and homing receptors has been observed in CIML-NK cells, enhancing their activation and anti-tumor capabilities (30, 31, 59). PM21-NK cells are already highly activated and cytotoxic (21, 23–25). CAP-NK cells, in addition to maintaining elevated expression of activating receptors such as CD16, NKG2D, and NKp46, also had increased expression of CD62L and FasL along with 4-1BB, indicating a highly active state. This enhanced activating receptor expression, along with improved metabolism, contributes to the CAP-NK-cells’ potent cytotoxicity *in vitro*, surpassing that of PM21-NK cells. Furthermore, CAP-NK cells exhibited memory-like properties with heightened responsiveness upon re-exposure to cytokines. Notably, CAP-NK cells displayed superior survival without cytokine support after resting and demonstrated increased longevity *in vivo*. Their ability to significantly reduce leukemia tumor burden in mice orthotopically injected with K562 cells underscores their effectiveness and bone marrow-homing capacity. These characteristics highlight the potential clinical value of CAP-NK cells for therapeutic applications.

Future directions in the development of NK cell-based therapeutics lay in various combinations with other agents such as therapeutic antibodies, BiKEs or TriKEs and NK cell engineering to arm them with CARs (60–62). Towards this future, CAP-NK cells have a very high and uniform expression of CD16 for potential use with therapeutic antibodies or CD16 targeting BiKEs (e.g., AFM13) as well as high expression of NKp46 for use with NKp46 -directed BiKEs (e.g., with ANKET). The robustness of the CAP expansion method also allows for incorporating genetic engineering to introduce CAR constructs or knock out inhibitory receptors (e.g. TIGIT) or antibody-targeted receptors that could result in NK cell fratricide (e.g. CD38, TIGIT) (63, 64).

In summary, the addition of cytokine activation to PM21-particle expansion induces the generation and expansion of memory-like NK cells and enhances the robustness of the PM21-particle platform while also improving the anti-tumor function of the final product. It also allows for in-process modifications and cryopreservation for off-the-shelf use. Overall, this study demonstrates that the CAP expansion platform can yield a superior NK cell product for manufacturing efficiency and potential therapeutic applications.

## Data availability statement

The RNA sequencing data presented in the study are deposited with links to BioProject accession number PRJNA1085873. All other original contributions presented in the study are included in the article/**Supplementary Material**, further inquiries can be directed to the corresponding author.

## Ethics statement

The studies involving humans were approved by University of Central Florida Institutional Biosafety Committee. The studies were conducted in accordance with the local legislation and institutional requirements. The human samples used in this study were acquired from a by- product of routine care or industry. Written informed consent for participation was not required from the participants or the participants’ legal guardians/next of kin in accordance with the national legislation and institutional requirements. The animal study was approved by University of Central Florida Institutional Animal Care and Use Committee. The study was conducted in accordance with the local legislation and institutional requirements.

## Author contributions

JO: Conceptualization, Formal analysis, Investigation, Methodology, Validation, Writing – original draft, Writing – review & editing. TC-P: Formal analysis, Investigation, Validation, Visualization, Writing – original draft, Writing – review & editing. MH: Investigation, Writing – review & editing, Validation, Data curation. JR-H: Investigation, Writing – review & editing, Validation. SG: Investigation, Writing – review & editing, Validation. JM: Investigation, Writing – review & editing, Validation. XZ: Formal analysis, Writing – review & editing. DA: Writing – review & editing, Validation, Methodology, Supervision. RI: Conceptualization, Methodology, Writing – original draft, Writing – review & editing, Validation. AC: Methodology, Project administration, Resources, Supervision, Writing – original draft, Writing – review & editing, Conceptualization, Validation, Funding acquisition.
