# Correction: PM21-particle stimulation augmented with cytokines enhances NK cell expansion and confers memory-like characteristics with enhanced survival

**DOI:** 10.3389/fimmu.2025.1650274

**Published:** 2025-07-04

**Authors:** Jeremiah L. Oyer, Tayler J. Croom-Perez, Md Faqrul Hasan, Javier A. Rivera-Huertas, Sarah B. Gitto, Joanna M. Mucha, Xiang Zhu, Deborah A. Altomare, Robert Y. Igarashi, Alicja J. Copik

**Affiliations:** Burnett School of Biomedical Science, College of Medicine, University of Central Florida, Orlando, FL, United States

**Keywords:** natural killer cell, NK cell therapy, memory-like NK cells, immunotherapy, adoptive cell therapy

In the published article, there was an error in the **Funding** statement. A funding source was mistakenly not acknowledged in the publication. The correct **Funding** statement appears below.

“The author(s) declare financial support was received for the research, authorship, and/or publication of this article. We would like to thank The Guillot-Henley Family AML Research Fund In Loving Memory of William L. Guillot fund, the FL DOH James and Ester King Program (Grant No. 9JK04), Bankhead-Coley Biomedical Research Program (3BN02, 4BB06) and the Live Like Bella Pediatric Cancer Research Initiative (22L04) as well as the University of Central Florida Preeminent Postdoctoral Program for funding. The authors declare that this study received funding from Kiadis Pharma, a Sanofi company. The funder was not involved in the study design, collection, analysis, interpretation of data, the writing of this article or the decision to submit it for publication.”

The authors apologize for this error and state that this does not change the scientific conclusions of the article in any way. The original article has been updated.

