# Supplementary material for: PM21-particle stimulation augmented with cytokines enhances NK cell expansion and confers memory-like characteristics with enhanced survival
Source: Front Immunol. 2024 Apr 22;15:1383281. doi: 10.3389/fimmu.2024.1383281 (PMC11070970; doi:10.3389/fimmu.2024.1383281)
Supplement: Supplementary file 1 [file DataSheet_1.pdf]

Supp. Table 1 Antibodies used in this study. The antibodies used for flow cytometry and their respective conjugate are shown.

Antigen	Clone	Conjugate	Source
41BB	4B4-1	PE-Dazzle™594	BioLegend
CD16	3G8	PE-Cy5	BioLegend
CD25	BC96	FITC	Biolegend
CD25	M-A251	PE	BioLegend
CD3	UCHT1	FITC	BioLegend
CD3	OKT3	PerCP-eF710	ThermoFisher
CD56	5.1H11	PE	BioLegend
CD56	5.1H11	AF®647	BioLegend
CD56	NCAM	APC/Fire™750	BioLegend
CD62L	DREG-56	PE/Cy7	Biolegend
CD96	NK92.39	PE	Biolegend
FASL	NOK-1	PE/Cy7	Biolegend
IFNγ	B27	PerCP-5.5	Biolegend
IL21R	2G1-K12	APC	Biolegend
Ki67	Ki-67	PE/Cy7	Biolegend
KIR2D	NFKBFS1	FITC	Miltenyi
NKG2D	1D11	APC	Biolegend
NKp46	9E2	PE-Dazzle™594	Biolegend
PVRIG	W16216D	APC	Biolegend
TIGIT	A15153G	PE/Cy7	Biolegend
TNFα	MAB11	PE-Dazzle™594	Biolegend

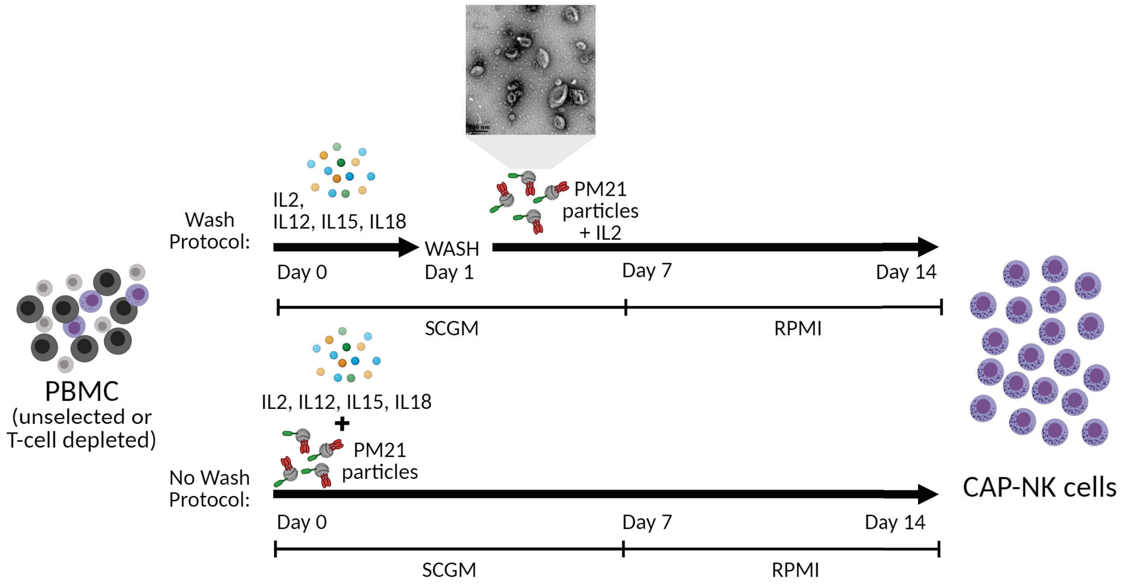

Supp. Figure 1. Schematic for CAP expansion protocols. Schematic of the NK cell expansion protocols used.

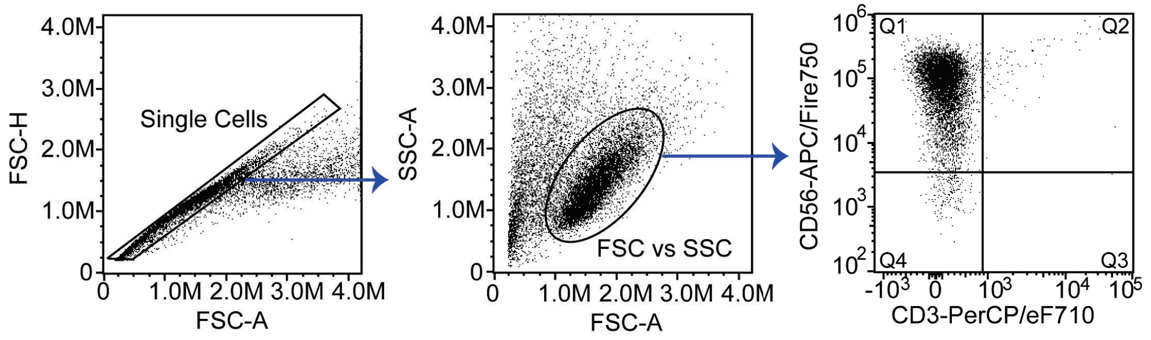

Supp. Figure 2. Example gating strategy for NK cells. Flow cytometry data were gated for NK cells by first using an FSC (area) vs FSC (height) dot plot gated on the single cell population ("single cells"). From the "single cells" population, an FSC vs SSC dot plot was created and gated on "lymphocytes". A CD56 vs CD3 dot plot was then created and gated on CD56^{+}CD3^{-} "NK cells" in Q1. An example gating strategy is shown.

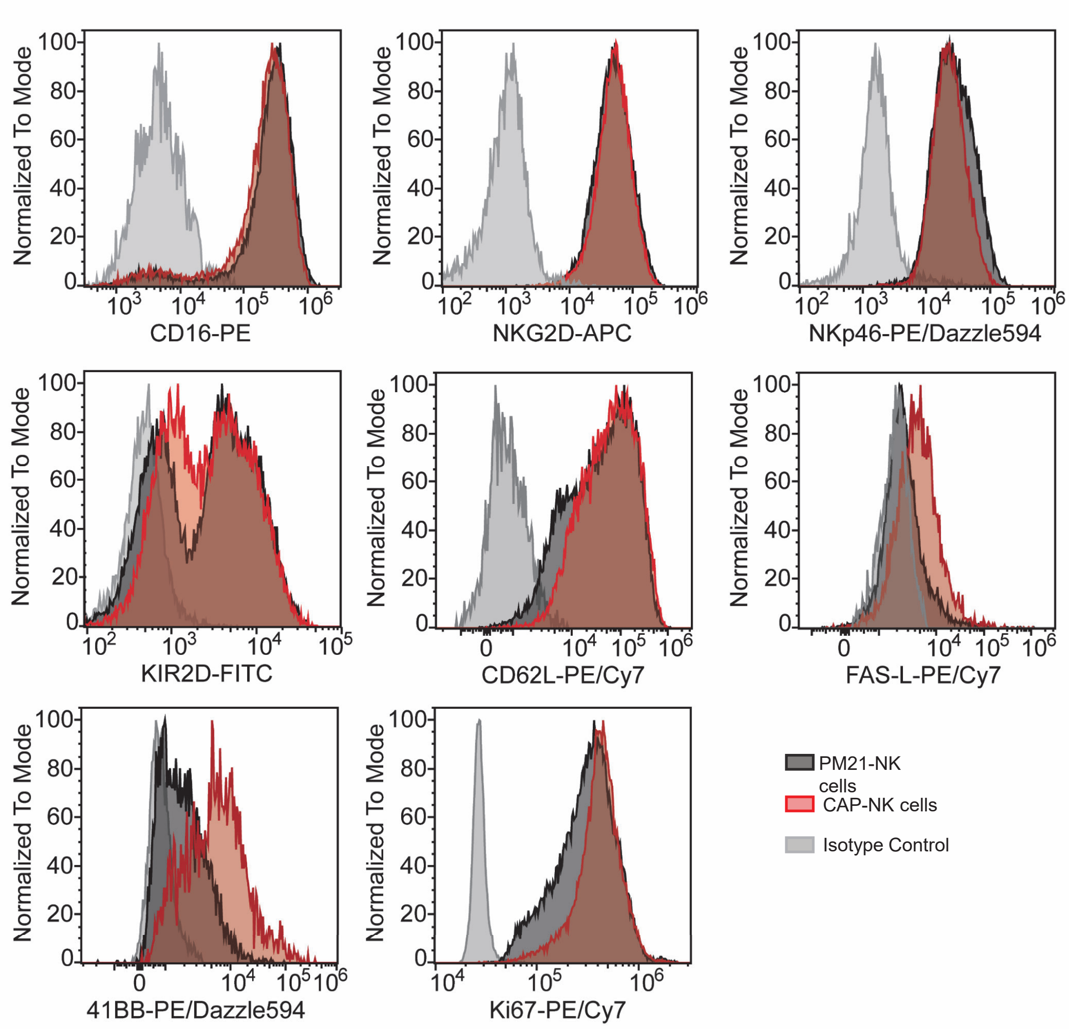

Supp. Figure 3. Histograms CAP-NK cell phenotype. PM21-NK cells and CAP-NK cells were analyzed for the presence of NK-cell receptors by flow cytometry. Representative histogram overlays are shown with isotype control in light gray and ligand-specific staining in black with gray fill for PM21-NK cells and red for CAP-NK cells.

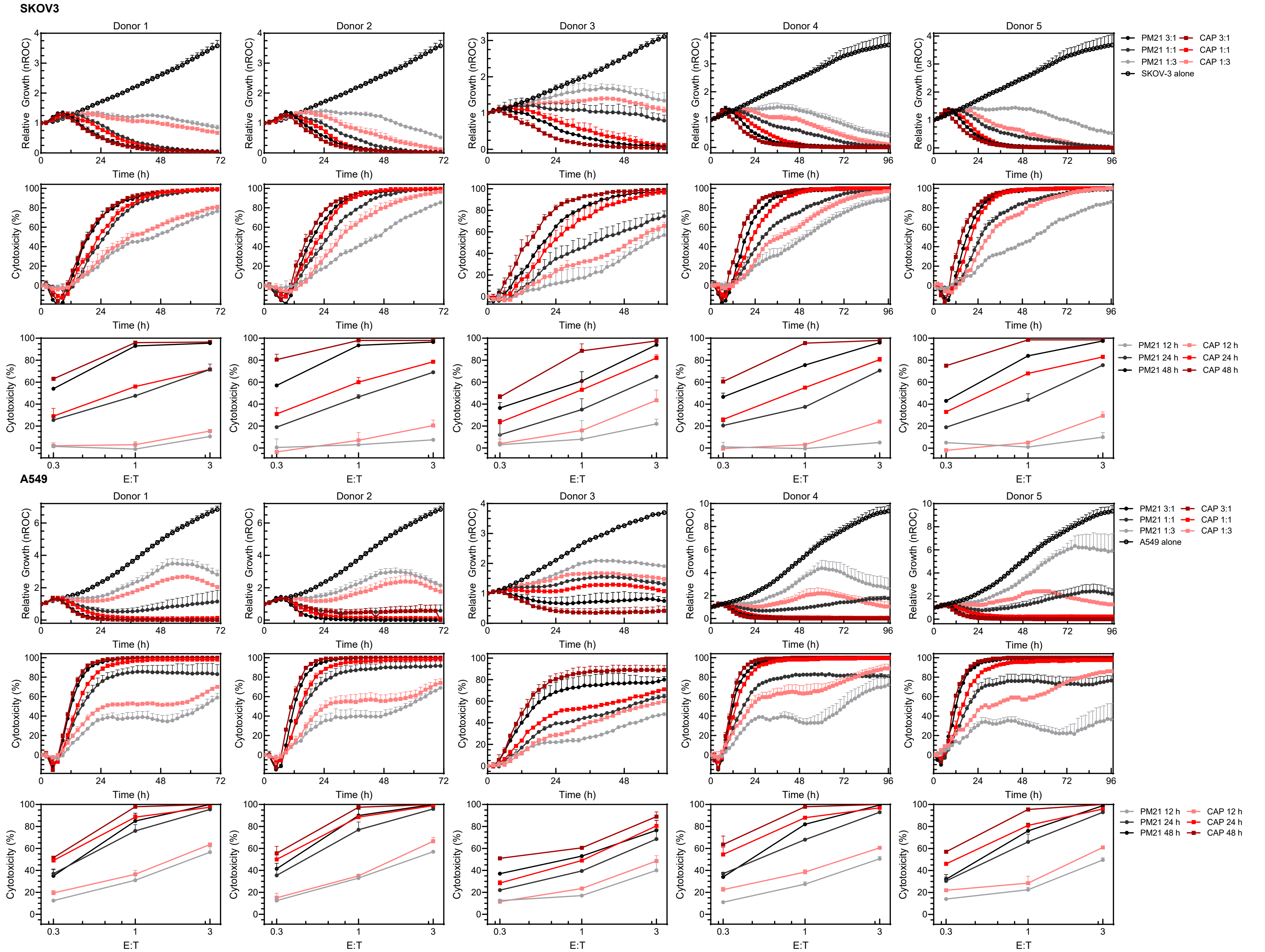

Supp. Figure 4. 3D Live-cell imaging cytotoxicity assay. Relative growth, cytotoxicity over time, and concentration-response curves from each donor are shown comparing the cytotoxicity of PM21-NK cells to CAP-NK cells at the E:T and times indicated against SKOV3 and A549 (

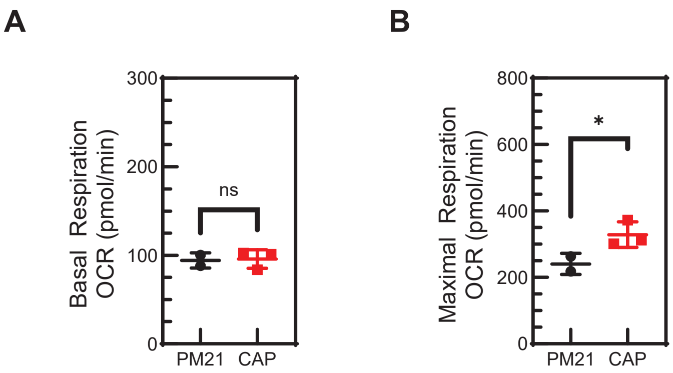

Supp. Figure 5. Oxygen consumption rate of second NK cell donor. T-cell-depleted PBMC were cultured with IL-2 and either PM21-particles alone or PM21-particles together with IL-12/15/18 (CAP) for 7-14 days. Metabolic profiles of PM21-NK cells (black circles) and CAP-NK cells (red squares) were measured by Seahorse (Agilent Technologies) on day 7 of culture. Oxygen consumption rate (OCR) profiles of NK cells from a second donor were determined from a Mito Stress Test Assay. Cumulative graphs comparing basal respiration (**A**) and maximal respiration (**B**) of PM21-NK cells and CAP-NK cells (measured in triplicate from one donor).

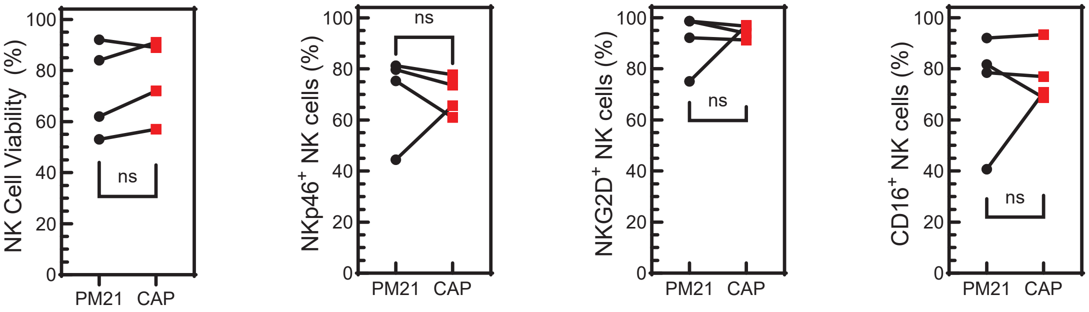

Supp. Figure 6. CAP-NK cells are amenable to cryopreservation. PM21-NK cells (black circles) and CAP-NK cells (red squares) were cryopreserved, thawed, and then analyzed for viability and expression of CD16 as well as activating receptors NKp46 and NKG2D by flow cytometry. No significant difference in viability or receptor expression was found between CAP-NK cells and PM21-NK cells.

Figure 9

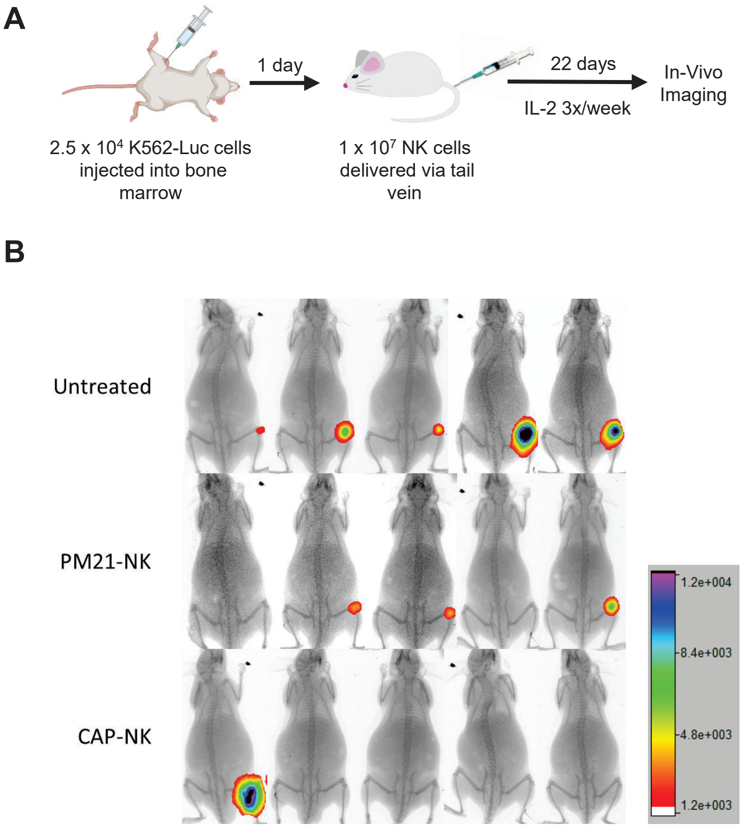

Supp. Figure 7. CAP-NK cells home to and diminish leukemia tumor burden in mice. A) Schematic depicting the orthotopic leukemia model in mice. K562-Luc cells (2.5 x 10^4 cells) were administered into the bone marrow of NSG mice via kneecap injections. One day later 1 x 10^7 NK cells were delivered via tail vein injection and IL-2 was administered 3x/week. **B**) In-vivo images were captured 22 days post-NK cell injection.
